# Divergent ECC1 effector homologs modulate host-specific virulence in cucurbit-infecting *Fusarium oxysporum*

**DOI:** 10.3389/fcimb.2025.1656785

**Published:** 2025-09-08

**Authors:** Babette V. Vlieger, Like Fokkens, Frank L. W. Takken, Martijn Rep

**Affiliations:** ^1^ Molecular Plant Pathology, University of Amsterdam, Amsterdam, Netherlands; ^2^ Laboratory of Phytopathology, Wageningen University & Research, Wageningen, Netherlands

**Keywords:** *Fusarium oxyporum*, effectors, host-specificity, CRISPR mutagenesis, plant pathogen

## Abstract

*Fusarium oxysporum* (Fo) is a soil-borne fungal pathogen that causes wilt disease in over one hundred plant species, with host-specific strains classified into *formae speciales* (ff. spp.). For example, Fo f. sp. *melonis* (Fom) only causes disease in melon while Fo f. sp. *radicis-cucumerinum* (Forc) can infect multiple cucurbit species. The virulence factors underlying host specificity in these cucurbit-infecting *formae speciales* have largely remained elusive, limiting our understanding of Fo-host interactions. A previous study identified Effector for Cucurbit Compatibility 1a (ECC1a), an avirulence protein from Fom that restricts cucumber infection when introduced into Forc. Here, we show that *ECC1a* is part of a previously unrecognized effector gene family, *ECC1*, abundantly present in strains that infect one or more cucurbit species. However, the role of this family in host compatibility is still poorly understood. Using gene knockout- and replacement strategies, we show that the *ECC1* gene family contributes to virulence of both Forc and Fom on cucumber and melon. Specifically, ECC1a contributes to Fom virulence on melon and Forc virulence on cucumber. ECC1b appears to be primarily involved in Fom virulence on melon.Expression profiling reveals a potential role of ECC1 during early stages of infection, suggesting involvement in initial host colonization. Together, these findings highlight the host- and *forma specialis*-specific functions of ECC1 homologs in Fo pathogenicity.

## 1 Introduction

Fusarium wilt disease, caused by the fungus *Fusarium oxysporum* (Fo), is a destructive plant disease on many economically important crops, including melon, cucumber, tomato, cotton, banana and soybean (Armstrong and Armstrong, 1981; Edel-Hermann and Lecomte, 2019). Within the Fo species complex, most pathogenic strains are highly host specific and grouped into *formae speciales* (f. sp.) based on their host range. For example, Fo f. sp. *cucumerinum* (Foc) is a major threat to cucumber (*Cucumis sativus)* (Vakalounakis et al., 2004) while *Fo*. f. sp. *melonis* (Fom) causes wilt disease in melon (*Cucumis melo*) (Oumouloud et al., 2013). Exceptionally, Fo f. sp. *radicis-cucumerinum* (Forc) infects three different species within the *Cucurbitaceae* (Edel-Hermann and Lecomte, 2019): cucumber, melon and watermelon (*Citrullus lanatus syn. C. vulgaris)*. Fom infection is typically associated with wilting, and can result in vascular browning, root rot and stem base decay (Seo and Kim, 2017; Edel-Hermann and Lecomte, 2019). Forc is best known for causing root and stem rot, often accompanied by wilting. Host pathogenicity of Forc and Fom is determined by so called ‘pathogenicity’ chromosomes (Van Dam et al., 2017; Li et al., 2020c): horizontal transfer of such a chromosome from Forc016, Fom001 or Fom005 to a non-pathogenic strain results in gain of virulence to cucurbits or melon, respectively (van Dam et al., 2016; Li et al., 2020c). From previous research, it has become clear that pathogenicity chromosomes carry many effectors that play a key role in host-specific virulence (Ma et al., 2010; Van Dam et al., 2017). These small *in planta* secreted proteins allow the pathogen to manipulate host processes to promote infection (Lo Presti et al., 2015). However, these effectors themselves can also be targets of plant immune receptors and elicit immune responses, in which case they are referred to as ‘avirulence (Avr) factors’ (Jones and Dangl, 2006). It is therefore hypothesized that a combination of presence and absence of certain (a)virulence genes on these chromosomes determines Fo host range (Li et al., 2020a).

The pathogenicity chromosomes of Forc016, Fom001 and Fom005 are highly similar, which enabled identification of potential effector genes that could contribute to a wide (cucurbits) versus a narrow host range (melon) of Fo (Li et al., 2021). Comparison of presence/absence variation and sequence differences among predicted effectors expressed *in planta*, revealed an effector candidate that is present in both Forc016 and Fom001 but with 15 amino acids difference in its protein sequence. Ectopic transformation of the Fom version of this gene (*g14035)* into Forc016 strongly reduced virulence on cucumber, while the ability of Forc16 to infect melon and watermelon was unaffected. This suggests that this effector is recognized in cucumber but not in its other hosts. This gene, which we will refer to as Effector for Cucurbit Compatibility 1a (*ECC1a*) is the first ‘non-host’ avirulence gene identified in Fo (Li et al., 2021).

**Figure 1:**
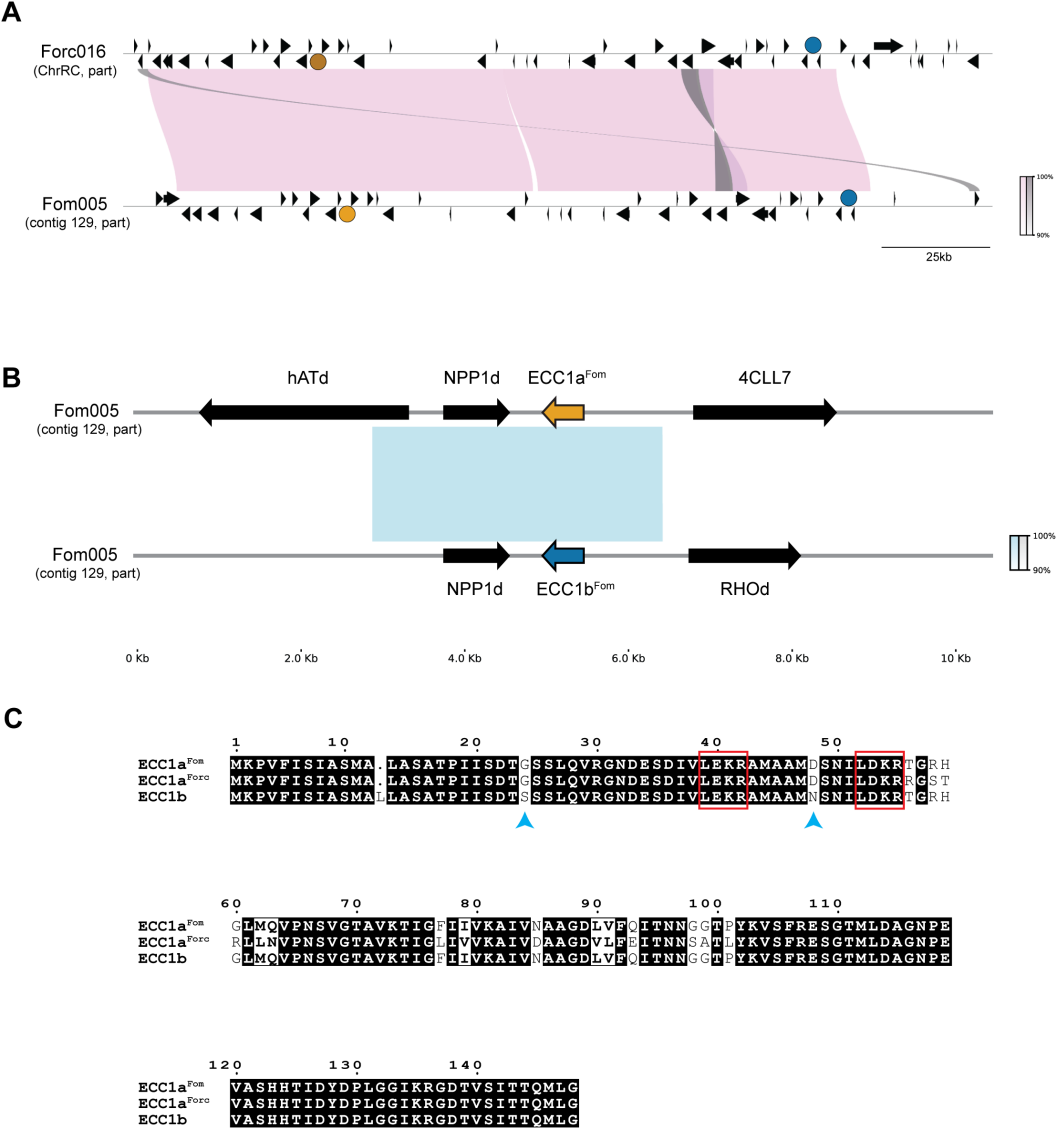
Comparative analysis of *ECC1* loci in pathogenicity chromosomes of Fom005 and Forc016. **(A)** Synteny plot showing alignment of a 200 kb region from the pathogenicity chromosomes of Fom005 and Forc016 containing the *ECC1a* (yellow) and *ECC1b* (teal) loci. Other open reading frames are indicated as black arrows. Pink blocks represent conserved regions (>99% identity), while grey blocks indicate inversions. **(B)** Detailed alignment of *ECC1* loci, including 5 kb upstream and downstream. The fact that *ECC1a* and *ECC1b* are part of a ~3.3 kb region with high nucleotide similarity (>99%) (represented by the blue block) indicates they probably result from a segmental duplication. This duplication also included *NPP1d*. **(C)** Protein alignment of ECC1a^Fom^, ECC1a^Forc^ and ECC1b. Conserved amino acids are shown with a black background; positions with differences are shown with a white background. Bold characters highlight amino acids with similar physicochemical properties. Potential Kex2 processing sites (LxxR motif) are highlighted with a red box. Amino acid differences between ECC1a and ECC1b are indicated with blue arrows.

While *ECC1a^Fom^* appears to limit virulence on cucumber, it remained unclear whether this gene contributes to virulence on melon. An ortholog in Forc, *ECC1a^Forc^*, is highly expressed during infection of cucumber and therefore a candidate virulence gene in this interaction (Li et al., 2021). Of note, both Fom001 and Forc016 carry an additional homolog of *ECC1a* that is identical in sequence between Fom and Forc (**Figure 1A**). This homolog will be referred to as *ECC1b* (previously *g14035–1 in* Fom001, and *g25*0–1 in Forc016). ECC1b differs by two amino acids from ECC1a^Fom^ (**Figures 1B, C**) (Li et al., 2021). *ECC1a* and *ECC1b* are located ~150 kb apart on a large, syntenic chromosomal region present in both isolates (**Figure 1A**). Here we aim to elucidate whether the two different *ECC1a* sequence types and/or *ECC1b* in *F. oxysporum* contribute to (a)virulence towards cucurbits. We used an RNP-based CRISPR-Cas9 approach to generate *ECC1a* and *ECC1b* single and double knockout mutants of Forc016 and Fom005. In addition, we have generated gene replacement strains in which *ECC1a^Fom^* is *in locus* replaced with *ECC1a^Forc^*, or *vice versa*, to determine how this gene replacement affects Fo host range.

## 2 Materials and methods

### 2.1 Chromosome synteny analysis

Comparative alignments of regions from the pathogenicity chromosomes of Fom005 and Forc016 were performed and visualized using PyGenomeViz v1.0.0 with default MUMmer alignment settings (Shimoyama, 2024). For this alignment, 200 kb segments were selected from each strain: 1,000,000 – 1,200,000 bp of the ChrRC of Forc016 (GenBank: CM008298.1) and 318,000 – 518,000 bp of contig 129 from Fom005 (GenBank: GCA_001703205.2). Gene annotation from Forc016 sequences were transferred to the corresponding Fom005 region by importing features in SnapGene v8.0.3 (https://www.snapgene.com), based on local sequence similarity. For the alignment of *ECC1a* (positions 38,884-49,385 bp) and *ECC1b* (positions 161,609-172,113 bp) loci from Fom005, the *ECC1b* region was reversed to account for orientation prior to visualization using the same PyGenomeViz settings. Other genes in these regions were further investigated by retrieving annotated gene names and functional description from the UniProt database (Bateman et al., 2025).

### 2.2 Protein sequence alignment and visualization

Protein sequence alignment was conducted using ClustalW through the Jalview platform v2.11.4.1 (Waterhouse et al., 2009). The resulting multiple sequence alignments were visualized using ESPript 3.0 (ENDscript – https://endscript.ibcp.fr) (Robert and Gouet, 2014).

### 2.3 Protein structure prediction and similarity analysis

The protein structures of ECC1 homologs were predicted using AlphaFold3 (Abramson et al., 2024). Structures were visualized and converted to PDB format using ChimeraX v1.10 (Meng et al., 2023). The resulting PDB file from ECC1a^Fom^ was used as input for structural similarity analysis with the DALI server (Holm, 2020). A pairwise structure comparison was performed against ToxA from *Pyrenophora tritici-repentis* (Sarma et al., 2005) (PDB entry: 1ZLD) and four known ToxA-like Fo effector structures: FOXGR_015533, SIX7, SIX8 and Avr2 (SIX3), for which PDB files were previously published (Yu et al., 2024).

### 2.4 Phylogenetic analyses

FOSC assemblies downloaded from GenBank are listed in **Supplementary Table S1**. Assemblies were selected based on having a high N50 where reference-guided assemblies were excluded, or based on being described as f. sp. *melonis*, *cucumerinum*, *niveum*, or *radicis-cucumerinum.* To infer a species phylogeny, we first identified BUSCO genes (busco version 5.7.1, with –metaeuk and hypocreales_odb10) and used a custom Python script to select BUSCO genes that were present as single-copy in all assemblies. These were aligned with Muscle (v 5.3.linux64) with default settings and trimmed with TrimAl (v1.5.rev0, -automated1: optimized for maximum likelihood inference). Subsequently, these trimmed alignments were concatenated with second custom Python script. We then used ModelFinder in IQTree to identify an optimal substitution model (GTR+F+I+R9) and IQtree with UFBoot (-m MFP -B 1000 -bnni -alrt 1000) to infer a consensus maximum likelihood phylogeny from this concatenated alignment with 1000 bootstrap replicates.

To infer the gene tree of *ECC1*, we used megablast with default settings to identify homologs of the gene sequences of *ECC1a^Fom^*, *ECC1a^For^* ^c^ and *ECC1b* (including introns) in the genome assemblies listed in **Supplementary Table S1**. We selected all hits with an E -value < 0.001 for which more than 50% of the query sequence was represented in the alignment. We used a one-line awk script to convert coordinates returned by BLAST to bed format, used *bedtools slop* to add 50 bp of flanking sequence where possible, merged overlapping regio with *bedtools merge* and used *bedtools getfasta* to obtain sequences in fasta format. We inferred a multiple sequence alignment with mafft (with –adjust direction accurately because sequences may be in different orientations). We inspected the resulting alignment and manually removed the 50 bp flanks in AliView (Larsson, 2014). We then inferred a gene tree with IQ-tree (Minh et al., 2020), implementing ModelFinder (Kalyaanamoorthy et al., 2017) to identify the best substitution model (K2P+G4), and UFBoot (Thi Hoang et al., 2017) for bootstrapping (1000 replicates).

### 2.5 Plant lines and fungal strains

Musk melon (*Cucumis melo* cv. ‘Cha-T’) and cucumber (*Cucumis sativus* cv. ‘Paraiso’) seeds were germinated and inoculated in a climate-controlled greenhouse at 25°C, with a relative humidity of 65% and 16/8h light/dark cycles.

*Fusarium oxysporum* (Fo) strains Fo *f.* sp. *radicis-cucumerinum* 016 (Forc016; ‘33’) (Lievens et al., 2007) and Fo f. sp. *melonis* 005 (Fom005; ‘Fom0123’) (Alvarez et al., 2005) were grown on Czapex Dox Agar (Difco) plates at 25°C in the dark.

### 2.6 Generation of donor DNA for ORF deletion and disruption strains

For ORF deletion mutants of the *ECC1* homologs, the ORF was replaced with a hygromycin (*HPH*)-*GFP* cassette. The HPH-GFP cassette was excised from pPK2-HPH-GFP (Michielse et al., 2008) using HindIII and EcoRV (ThermoFisher Fermentas). Homologous flanking regions of 1kb upstream (primers FP10575 and FP10576) and downstream (primers FP10577 and FP10578) of the ORF were inserted adjacent to the cassette using Gibson cloning (NEBuilder^®^ HiFi DNA Assembly Cloning Kit, New England Biolabs (UK) Ltd.). The resulting constructs are referred to as pΔECC1aFom-hphGFP, pΔECC1aForc-hphGFP, and pΔECC1b-hphGFP. Primers are listed in **Supplementary Table S2**.

For CRISPR/Cas9-mediated ORF disruption, the same HPH-GFP cassette was used, flanked by homologous regions of 400bp around the targeted double-strand break site within the ORF. These regions were amplified using primers FP10856 and FP10857 and primers FP10858 and FP10859. The constructs were named pCRISPRΔECC1aFom, pCRISPRΔECC1aForc, and pCRISPRΔECC1b.

### 2.7 Generation of donor DNA for complementation and gene replacement strains

For ectopic complementation of *ECC1* homologs, constructs were generated by Gibson cloning the promoter region (~1kb upstream of ORF), the ORF and the terminator (~400 bp downstream of ORF) upstream of a phleomycin cassette in pRW1p (Houterman et al., 2008), digested by EcoRI and HindIII (ThermoFisher Fermentas). An additional ~600bp homologous flanking region downstream of the ORF was inserted downstream of the cassette to facilitate homologous recombination. The terminator was duplicated since it could be a shared terminator for both the *ECC1* gene and the neighboring predicted gene encoding a NPP1 domain-containing protein (*NPP1d*) (**Figure 1**). The resulting constructs were named pECT-ECC1aFom-phleo, pECT-ECC1aForc-phleo, and pECT-ECC1b-phleo. Assembly was done using the following primer sets (**Supplementary Table S2**): primers used for introducing the upstream and downstream region are FP11990+FP11991 and FP11992+FP11993 for *ECC1a^Fom^*, FP11990+FP11991 and FP11992+FP11994 for *ECC1a^Forc^* and FP11995+FP11996 and FP11992+FP11993 for *ECC1b*.

To enable *in locus* complementation, pBV1n was generated by replacing the phleomycin cassette in pRW1p with a nourseothricin resistance cassette amplified from pZPnat1 (GenBank AY631958.1), using FP12743+FP12744 and FP12756+FP12757 and assembled by Gibson cloning. This vector served as a backbone for generating pLOC-ECC1aFom-nat, pLOC-ECC1aForc-nat, and pLOC-ECC1b-nat. Inserts containing the promoter (~1kb upstream of ORF), the ORF and the terminator (~ 400 bp downstream of ORF) were cloned upstream of the cassette, while a 600bp downstream flanking region was inserted downstream, using FP12739+FP12742 and FP12745+FP12746 for *ECC1a^Fom^*, FP12739+FP12742 and FP12745+FP12747 for *ECC1a^Forc^* and FP12741+FP12742 and FP12745+FP12746 for *ECC1b*.

### 2.8 Cas9 production and purification

The pHis-parallel1-_NLS_Cas9 (Addgene Catalog #112065) plasmid was used to express Cas9 in BL21 GOLD (DE3) cells as described (Pokhrel et al., 2022). Protein expression was induced with 0.3 mM IPTG at 25°C for 18 hours. For purification, BL21 GOLD cells were resuspended in Cas9 Lysis Buffer (20 mM Tris–HCl, 300 mM NaCl, 10 mM imidazole, 1 mM PMSF, pH 8.0) containing ~0.1 mg/mL Lysozyme (Sigma-Aldrich) and incubated for 1h at 4°C. Cells were lysed by disrupting them four times with a French Press (ThermoFisher Scientific). The lysate was clarified by centrifugation at 50,000 x g for 1 hour at 4°C using a Beckman Coulter Avanti J-E centrifuge equipped with JA 25.50 rotor. Recombinant Cas9 was purified using ÄKTA (Cytiva) with His Trap FF (5 mL) column (Cytiva). The columns were washed with Cas9 Wash Buffer (20 mM Tris–HCl, 500 mM NaCl, 25 mM imidazole, pH 8.0) and proteins were eluted using Cas9 Elution Buffer (20 mM Tris–HCl, 500 mM NaCl, 500 mM imidazole, pH 8.0). Proteins were concentrated using Amicon Centriplus Centrifugal Filter Devices YM-100 (Millipore, 100 kDa cut-off). Amicon Ultra – 4 Centrifugal Filters Ultracel – 50K (Millipore, 50kDa cut-off) were used for the final concentrating steps. For buffer exchange, PD10 desalting columns (Cytiva) were used. Recombinant Cas9 was stored in Cas9 storage buffer (20 mM HEPES, 300 mM NaCl, 10% glycerol, pH 7.5).

### 2.9 *In vitro* transcription of sgRNA

sgRNA were screened for potential off-target sites using the CRISPR gRNA Design Software from Geneious v2023.2 (https://www.geneious.com) and blastn (megablast, default options, NCBI). sgRNAs were generated using the New England Biolabs EnGen^®^ sgRNA Synthesis Kit (*S. pyogenes*) as described (Pokhrel et al., 2022). Oligos generated for the *in vitro* RNA synthesis are given in **Supplementary Table S3**.

### 2.10 *In vitro* cleavage assay

sgRNA cleavage efficiency was checked by *in vitro* cleavage assay as described (Pokhrel et al., 2022), with some minor alterations in the mastermix. The mastermix consisted of 1x Cas9 nuclease buffer (20 mM HEPES, 100 mM NaCl, 5 mM MgCl_2_, and 0.1 mM EDTA, pH 6.5), 0.5 μg sgRNA, 0.5 μg Cas9, 100 ng of DNA template and DEPC water to a final volume of 20 μL. Reactions were incubated for 1 hour at 37°C. After incubation, samples were treated with Proteinase K (ThermoFisher Scientific) for 10 minutes at 37°C. The cleavage activity was visualized by gel electrophoresis on a 0.8-1% agarose gel.

### 2.11 Fo tissue culture and protoplast isolation

Protoplast isolation was performed using a protocol based on methods previously described (Brückner et al., 1992; Tudzynski et al., 1999; Janevska et al., 2018). Briefly, the Fo pre-cultures were prepared using 100 mL of Darken medium (87 mM sucrose, 7.6 mM (NH_4_)_2_SO_4_, 0.7 g/100 mL CaCO_3_, 15 g/L corn steep solids) and used to inoculate 100 mL ICI main culture medium (403 mM D-glucose•H_2_O, 5.9 mM MgSO_4_ • 7 H_2_O, 3.6 mM KH_2_PO_4_, trace elements (1:500; 36 mM FeSO_4_•7 H_2_O, 0.6 mM CuSO_4_•5 H_2_O, 5.6 mM ZnSO_4_•7 H_2_O, 0.6 mM MnSO_4_• H_2_O, 0.08 mM (NH_4_)_6_Mo_7_O_24_•4 H_2_O), 5.6 µM L-glutamine) with 0.5% (v/v) pre-culture grown for 3 days at 150 rpm and 25°C (Janevska et al., 2018). Young mycelium was harvested using Miracloth, washed with sterilized MQ and KCl/CaCl_2_ Buffer (1.2 M KCl, 50 mM CaCl_2_•2 H_2_O), and treated with enzyme solution (4 g/L Lysing enzymes (Sigma-Aldrich), 0.2 g/L Lyticase (Sigma-Aldrich), 0.2 g/L Yatalase (Takara), 0.2 g/L Albumin Fraktion V (Merck) dissolved in KCl/CaCl_2_ buffer). After filtration using glass filters (VitraPOR Por. 1/2, ROBU) and centrifugation, protoplasts were washed and resuspended in 1x STC buffer (1.2 M sorbitol, 10 mM Tris, 50 mM CaCl_2_, pH 7.5) (Brückner et al., 1992). The protoplasts were then diluted to a final concentration of 2 × 10^7^ protoplasts/mL.

### 2.12 Fo transformation with RNPs

Fo protoplast transformation was performed using a protocol based on methods previously described (Brückner et al., 1992; Tudzynski et al., 1999; Janevska et al., 2018; Pokhrel et al., 2022). Per transformation, RNPs were assembled in a 50 µL reaction containing 1x Cas9 nuclease buffer, 20µg recombinant Cas9 and 20µg sgRNA. The mix was incubated at 37°C for 20 min. The 1:1 Cas9:sgRNA ratio was previously determined by the *in vitro* nuclease assay as optimal. Per transformation, 200 µl protoplasts were mixed with 50 µL of the RNPs and 300–400 ng amplified donor DNA, and incubated for 20 min at RT. The RNP mixture was transferred to 1.6 mL PEG solution (50% w/v PEG 4000, 50mM CaCl_2_, 10 mM Tris, pH 7.5) (Brückner et al., 1992). After an incubation of 10 min at RT, the reaction was terminated by adding 3.2 mL 1xSTC. The transformation mixture was mixed with Regeneration Medium (RM) (700 mM sucrose, 0.5 g/L yeast extract, 20 g/L agar) and incubated O/N at RT before adding the selective layer containing antibiotics to a final concentration of 100-150 µg/mL hygromycin (Duchefa Biochemie) for the knockout mutants or 50 µg/mL nourseothricin (Jena Bioscience) or 100 µg/mL zeocin (InvivoGen) for the complementation and gene replacement strains. Edits at *ECC1* loci were verified by PCR analysis (**Supplementary Figures S2**, **S3**) and Sanger sequencing.

### 2.13 Agrobacterium-mediated Fo transformation

*Agrobacterium tumefaciens*-mediated Fo transformation was used to obtain ectopic complementation and gene replacement strains in Fom005 and Forc016 as described previously (Takken et al., 2004; Michielse et al., 2008). Monosporic isolates of transformants were obtained on Potato Dextrose Agar (Difco) as described (Li et al., 2020b).

### 2.14 Fo disease assays

To test the virulence of the Fo transformants, melon seedlings (nine days old) and cucumber seedlings (seven days old) were inoculated with water (mock), Fom005 (WT), Forc016 (WT) or the *ECC1* mutants at 25°C. The plants were inoculated with 10^7^ spores/mL via the root dip method described previously (Rep et al., 2004). Spores were collected of five-day-old Fusarium cultures grown in liquid NO_3_ medium (0.17% yeast nitrogen base, 3% sucrose, 100mM KNO_3_). The number of plants per treatment varied per plant species per replicate and is specified in the corresponding figure legends. Disease progression was assessed 14 days post inoculation (dpi) by measuring plant fresh weight and scoring disease severity using a disease index ranging from 0-4, where 0 indicated no symptoms; 1, slight discoloration (browning)/root rot symptoms, only at tip of main root; 2, discoloration or root rot symptoms and stem lesions visible aboveground, growth distortion; 3, very clear root rot symptoms of the entire root system, often with a large lesion extending above the cotyledons, severe growth distortion and wilting; 4, plant either dead or very small and wilted (**Supplementary Figure S4**).

Statistical analyses were performed in R version 4.4.2 (R Core Team, 2024). Normality was assessed using the Shapiro-Wilk test, which indicated that data did not follow a normal distribution. Fresh weight data were analyzed using Kruskal-Wallis tests, followed by Dunn’s *post hoc* test with Benjamini-Hochberg correction for multiple testing. Disease severity scores were assessed using Mann-Whitney U tests with Benjamini-Hochberg correction.

### 2.15 Fo transcriptome sampling

Ten-day-old melon seedlings and seven-day-old cucumber seedlings were inoculated using the root-dip method as described above, with a modification: to allow sufficient tissue collection at early timepoints, the roots were trimmed to approximately 2 cm (instead of 1 cm), prior to inoculation with wild-type Fom005, Forc016 or Milli-Q (mock treatment). After inoculation, the seedlings were potted in vermiculite supplemented with nutrients. The roots of three seedlings were harvested per replicate at 2-, 4-, 7- and 10-days post-inoculation (dpi), flash-frozen in liquid nitrogen and subsequently freeze-dried.

### 2.16 RNA extraction

Freeze-dried infected root material was disrupted using 4 mm metal beads in a tissue lyser (Qiagen) at 30 Hz for 2 min. The entire root system was used as input for RNA extraction. RNA was extracted using the RNeasy Plant Mini Kit (Qiagen) including Appendix D: Optional On-Column DNase Digestion with the RNase-Free DNase Set (Qiagen). An additional DNase treatment was performed with RNase-free DNase I (ThermoFisher Scientific) according to the manufacturer’s instructions. Then, 1 µg of total RNA was used for cDNA synthesis by RevertAid H Minus Reverse Transcriptase (ThermoFisher Scientific) following the manufacturer’s protocol.

### 2.17 TaqMan real-time PCR assays

Probes and primers were designed using IDT PrimerQuest™ Tool (Owczarzy et al., 2008) and Primer3Plus version: 3.3.0 (Untergasser et al., 2012). TaqMan assays were performed on a QuantStudio 3 Real-Time PCR System (ThermoFisher Scientific). The 10 µL reactions contained (final concentration): 0.25 U of DreamTaq DNA Polymerase (ThermoFisher Scientific), 1x DreamTaq buffer, 2 pmol of each probe, 5 pmol of each primer, 0.2 mM dNTPs (each), 1 µL of cDNA. Multiplex reactions were performed for targets *ECC1a* and *ECC1b*, whereas *EF1α* reactions were run separately (simplex). The TaqMan RT PCR program was set as follows: 2 min at 95°C; 45 cycles of [15 s at 95°C, 48 s at 68°C, 12 s at 68°C (data collection)]. Each sample was run in three technical replicates. A no-template control (NTC), where Milli-Q replaced the template, was included, as well as mock-inoculated plant samples as a second negative control. Primers and probes are listed in **Supplementary Table S5**.

## 3 Results

### 3.1 *ECC1a* is part of a large effector family

Previous analyses have shown that *ECC1a* is located 150 kb upstream of a homolog, *ECC1b* (Li et al., 2021). *ECC1b* is highly similar to *ECC1a^Fom^*, and an identical homolog is present in Forc016. Synteny in this entire region is highly conserved between Fom and Forc (**Figure 1A**). *ECC1a* and *ECC1b* are part of a ~3.3 kb segmental duplication, which also includes a gene that encodes a secreted protein with a necrosis inducing protein (NPP1)-like domain, which we here call *NPP1d*. To determine whether *ECC1a* and *ECC1b* are part of a larger family and whether they are also present in other cucurbit-infecting isolates, a dataset of 149 *Fo* genome assemblies was compiled. Of these, 99 are from strains that are known to, or predicted to, infect a member of the cucurbits (**Supplementary Table S1**) while the other 50 are from strains that are pathogenic on other plant species or non-pathogenic isolates isolated from soil or asymptomatic hosts. We inferred a phylogeny for the strains in this dataset, and observed, consistent with previous analyses (Sabahi et al., 2021), that f. sp. *melonis*, f. sp. *cucumerinum* and f. sp. *niveum* are polyphyletic, i.e. members of the same *forma specialis* cluster in different clade in the phylogeny (**Supplementary Figure S1**).

**Figure 2:**
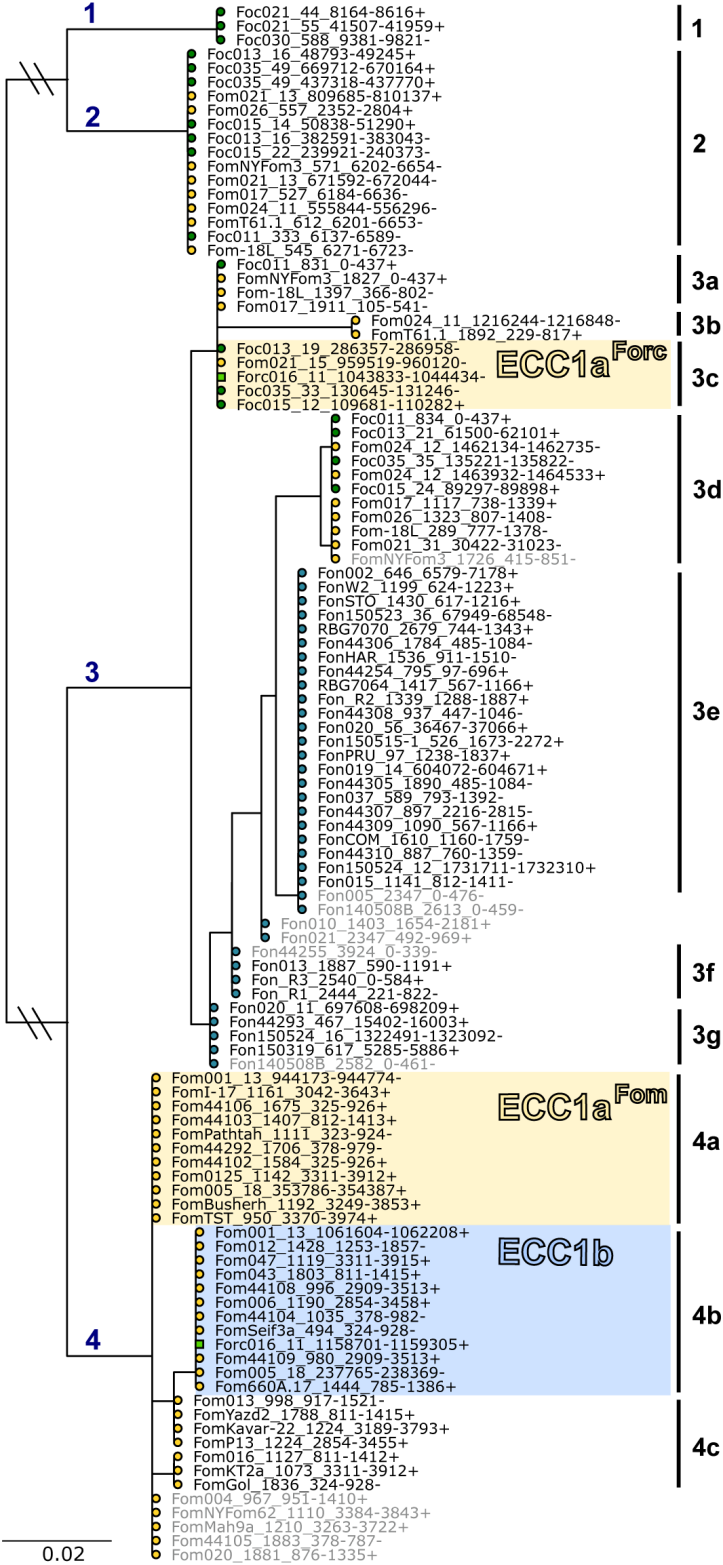
ECC1 is part of a large effector family that is specific to cucurbit-infecting strains. Gene tree with homologs of *ECC1a^Fom^*, *ECC1a^Forc^* and *ECC1b*, where leaves are shaped and colored according to host range (circles: yellow – melon, dark green – cucumber, blue – watermelon, green square – melon, watermelon and cucumber). This family can be divided into four subfamilies (indicated with blue numbers above branches) and includes 13 distinct genotypes (indicated with subfamily index + letter); genotypes of *ECC1a^Fom^*, and *ECC1a^Forc^* are highlighted in yellow and *ECC1b* in blue. Sequences that are partial hits due to contigs breaks or assembly gaps are indicated in grey. Long branches separating subfamilies 1 and 2 from subfamilies 3 and 4 have been shortened to improve overall visibility. This is indicated with two small diagonal lines through the respective branches.

We then searched for *ECC1* homologs in our dataset with BLAST and found that *ECC1a* is part of a family of effectors that is present in many, but not all, strains that infect melon, watermelon and/or cucumber. By inferring a gene tree of the ECC1 gene family, it was found that *ECC1* homologs can be grouped into four subfamilies, where *ECC1a^Fom^* and *ECC1b* belong to the same subfamily, but *ECC1a^Forc^* does not (**Figure 2**). Not counting sequences that are disrupted by assembly errors (i.e. located at start or end of contigs, or interrupted by an assembly gap), these four subfamilies could be further subdivided into 13 different sequence types: clades in which the phylogenetic distance between members is zero (indicated with number 1-4c in **Figure 2**). Some sequence types are specific to a single *forma specialis*. Subfamily 4, which includes *ECC1a^Fom^* and *ECC1b*, is present only in f. sp. *melonis*, except for the *ECC1b* gene that is present in Forc016. In contrast, subfamily 2, that includes *ECC1a^Forc^*, is present in f. sp. *melonis*, *niveum* and *cucumerinum*, and both f. sp. *melonis* and f. sp. *cucumerinum* strains carry the exact *ECC1a^Forc^* genotype. Surprisingly, no copy of *ECC1a^Forc^* or *ECC1b* was found in the assemblies of Forc031 and Forc024, while these are very closely related to Forc016 and have the same host range. Closer inspection revealed that these assemblies carry partial copies of *ECC1a^Forc^*/*ECC1b* that correspond to the parts that are identical between these genes, interrupted by a gap in the assembly. This suggests that detection of *ECC1* failed due to an assembly error: collapse of the 3.3 kb segmental duplication that *ECC1a* and *ECC1b* are located on. Notably, strains that share an *ECC1* sequence type are not necessarily phylogenetically closely related, suggesting that these genes have transferred horizontally between cucurbit-infecting strains (**Figure 2**; **Supplementary Figure S1**). Based on these phylogenetic analyses, we predict that ECC1a^Fom^ and ECC1b are important for infection of melon, given the fact that they are present in most melon-infecting strains in our dataset. Moreover, we predict that ECC1a^Forc^ may contribute to virulence towards melon and cucumber, since it is present in multiple distinct lineages that group into these *formae speciales*, and it is highly expressed in Forc016 during infection of cucumber (Van Dam et al., 2017; Li et al., 2021).

### 3.2 ECC1 proteins are structurally similar to ToxA-like effectors

**Figure 3:**
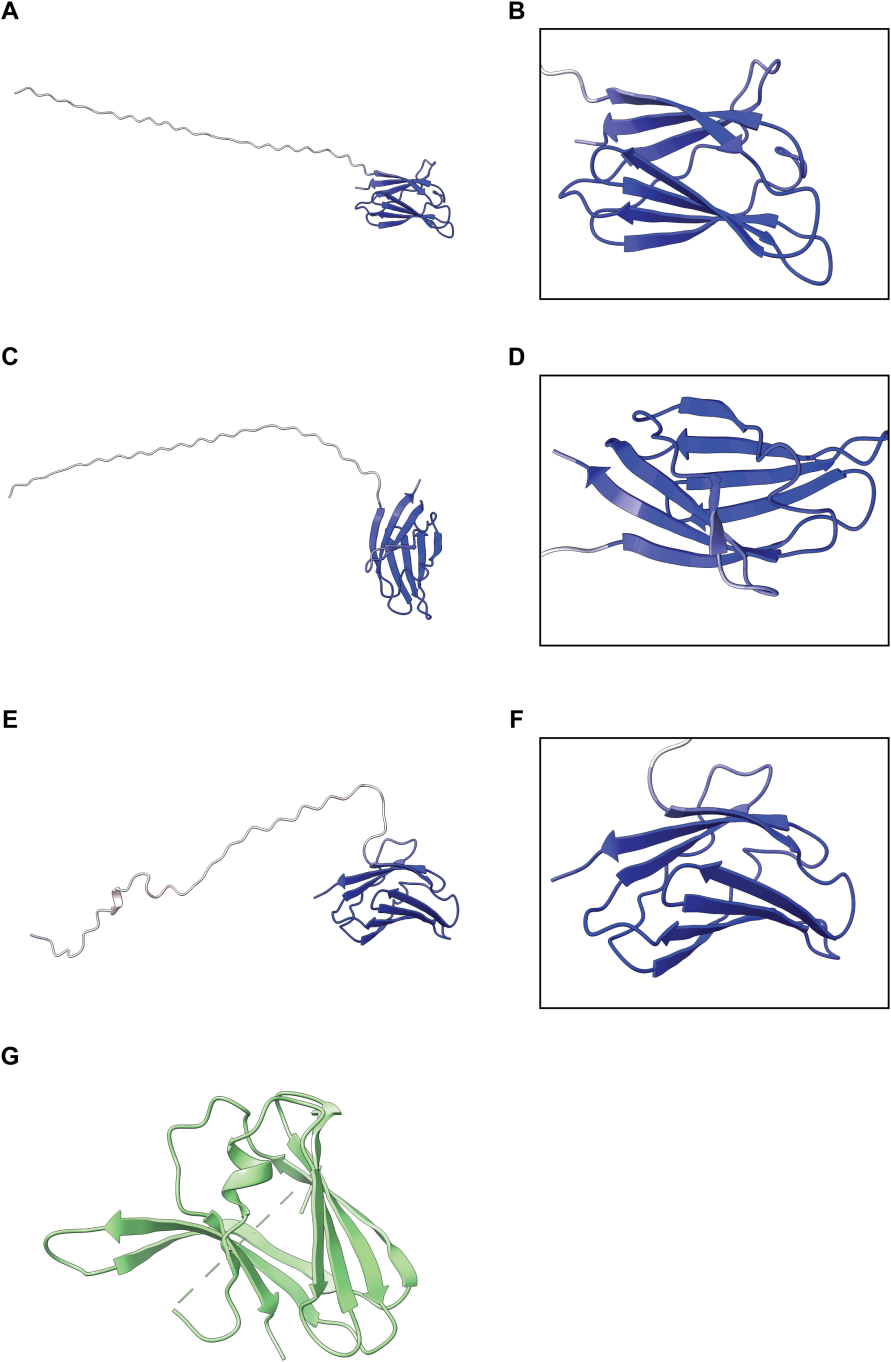
Predicted structures of ECC1 homologs. Alphafold3 models of ECC1a^Fom^ **(A, B)**, ECC1a^Forc^ **(C, D)** and ECC1b **(E, F)** show full-length structures. Right panels **(B, D, F)** display zoom-in views of the C-terminal β-sheet “sandwich”. All models are colored by per-residue pLDDT confidence score (blue = high confidence, red = low confidence). **(G)** Crystal structure of ToxA from *Pyrenophora tritici-repentis* (Sarma et al., 2005) (PDB entry: 1ZLD).

To obtain more information on the potential function of ECC1 homologs, protein structure predictions were generated for all three ECC1 homologs using AlphaFold3 (**Figure 3**). The resulting models revealed a β-sandwich fold, which is a characteristic structural feature of ToxA-like effectors. Structural similarity between ECC1a^Fom^ and ToxA (from *Pyrenophora tritici-repentis*), as well as known ToxA-like Fo effectors (FOXGR_015533, SIX7, SIX8 and Avr2 (SIX3)) was assessed using the DALI server resulting in Z-scores of 7.4 (ToxA), 7.5, 6.8, 6.6 and 5.5, respectively. Since Z-scores between 2 and 8 are generally indicative of structural homology (Holm, 2020), these results suggest that ECC1^Fom^ is structurally related to the ToxA and the ToxA-like effector family. Interestingly, when reviewing the protein sequence alignment (**Figure 1C**), potential Kex2 processing sites (LxxR motif) were found in the sequence of the three homologs. Kex2 sites have been found before in fungal ToxA-like effectors and in other effectors from Fo (Outram et al., 2021). These results suggest that ECC1 may be part of the ToxA-like effector family and could be Kex2 pro-domain-processed (K2PP).

### 3.3 Increased recombination efficiency on Fo pathogenicity chromosomes with CRISPR/Cas9 mutagenesis

To study the function of ECC1a^Fom^, ECC1a^Forc^ and ECC1b in different genetic backgrounds, single- and double knockout and complementation strains were generated, and strains in which a knockout of *ECC1a* was complemented with the *ECC1a* gene from the other *forma specialis*. Initially, using PEG-mediated protoplast transformation, *ECC1* open reading frame (ORF) deletion mutants were generated in Fom005 and Forc016. From this transformation event only three out of the 57 transformants screened contained the desired ORF deletion (**Supplementary Table S4**). To increase efficiency, we adapted an RNP-based CRISPR-Cas9 approach to generate ORF disruption mutants in Fom005 and Forc016. In addition, this strategy enabled efficient generation of double knockout mutants. An overview of the mutants is given in **Supplementary Table S4**.

Next, to verify that any observed phenotypes in the knockout mutants are caused by the respective gene deletions, we also generated complementation strains of the single knockout mutants by reintroducing the native gene either at the original locus or ectopically. To generate *in locus* complementation strains (complemented with the endogenous native gene) and gene replacement strains (complemented with the homolog from the other *forma specialis*), we again employed the CRISPR/Cas9 system. Ectopic complementation and gene replacement strains were generated via *Agrobacterium tumefaciens*-mediated transformation (ATMT). Together, for each type of single knockout, two to four complementation or gene replacement strains were generated. Finally, we tested the virulence of all these mutants towards melon and cucumber in disease assays.

### 3.4 *ECC1a^Forc^* contributes to virulence towards cucumber in Forc016 but does not expand the host range of Fom005

**Figure 4:**
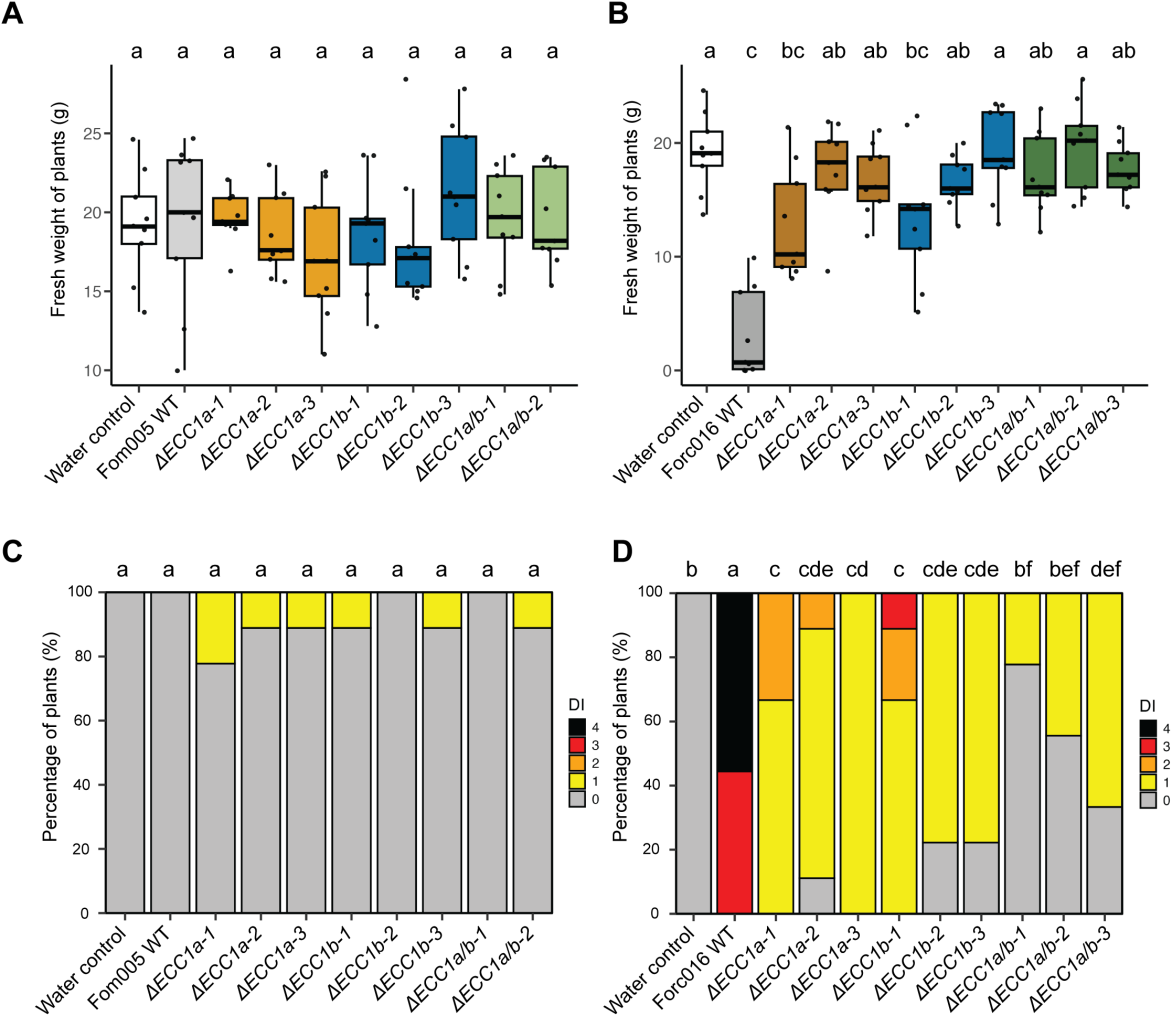
Knockout of *ECC1a* and *ECC1b* in Forc016 reduces virulence towards cucumber. Seven-day-old cucumber (*Cucumis sativus* cv. *Paraiso*) seedlings were inoculated with water (mock), WT, *ΔECC1a, ΔECC1b* and *ΔECC1a/b* knockout mutants of Fom005 **(A, C)** or Forc016 (**B, D**) (n=10) at 25°C. **(A, B)** Plant fresh weight (FW) was measured (in grams) 14 days post inoculation. **(C, D)** Disease symptoms were scored 14 days post inoculation. Means followed by a common letter are not significantly different by Kruskal-Wallis with Dunn’s *post hoc* test and Benjamini-Hochberg correction for FW **(A, B)** or Mann-Whitney U test with Benjamini-Hochberg correction for disease symptoms **(C, D)** at the 5% level of significance.

To investigate whether ECC1a or ECC1b impact virulence of Fom on cucumber by Fom, we compared the virulence towards cucumber of knockout mutants in Fom005 with that of the wild- type strains. No obvious differences in growth or colony morphology were observed for the *ECC1* deletion mutants under standard *in vitro* culture conditions. Virulence was quantified by scoring disease severity (**Supplementary Figure S4**) and measuring fresh weight, and representative pictures of the plants were taken (**Supplementary Figures S5**–**S8**). Overall, there were no significant differences in disease severity between cucumber plants inoculated with ORF deletion mutants and ORF disruption mutants (**Supplementary Table S4**). As expected, wild-type Fom005 was non-pathogenic on this host (**Figures 4A, C**). As *ECC1a^Fom^* acted as a non-host avirulence gene in Forc016 (Li et al., 2021), we reasoned that deletion of *ECC1a^Fom^* in Fom may result in an increase in virulence towards cucumber. However, neither the single nor the double knockout mutants of Fom005 showed a significant reduction in plant fresh weight or disease symptoms on this plant species (**Figures 4A, C**). Together, these results indicate that ECC1 homologs alone are not sufficient to restrict the host range of Fom.

To determine whether ECC1a^Forc^ and ECC1b play a role in virulence of Forc016 towards cucumber, the same setup was used to test the impact of single and double knockout mutants on disease. As anticipated, wild-type Forc016 caused severe disease symptoms and significantly reduced fresh weight in cucumber plants compared to mock (**Figures 4B, D**). In contrast to results observed in Fom005, fresh weight of plants inoculated with *ECC1a^Forc^* or *ECC1b* single and double knockout mutants significantly differed from those observed for the Forc016 wild type. Moreover, disease symptoms were consistently significantly less severe in all knockout mutants relative to those of the wild-type strain. The *ECC1* double knockout mutants did not consistently show a larger reduction in virulence on cucumber as compared to the single knockout mutants. Together, these results suggest that both ECC1a^Forc^ and ECC1b contribute to cucumber infection by Forc016.

**Figure 5:**
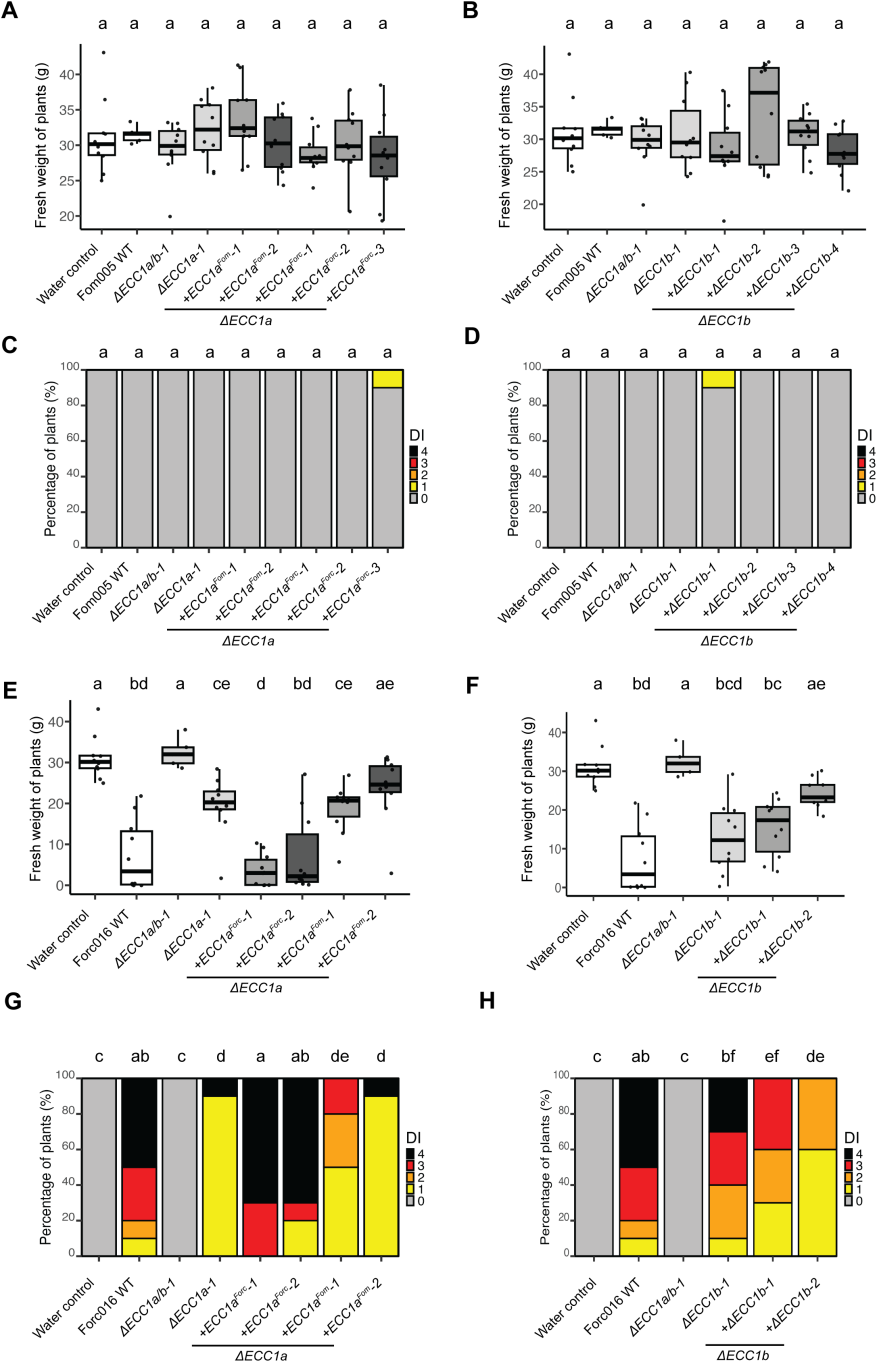
Complementation of *ECC1* partially restores virulence of Forc on cucumber and reveals host-specific roles. Seven-day-old cucumber (*Cucumis sativus* cv. *Paraiso*) seedlings were inoculated with water (mock), WT, *ΔECC1a*, *ΔECC1b*, *ΔECC1a/b* knockout mutants, complementation and gene replacement strains of Fom005 **(A-D)** or Forc016 **(E-H)** (n=10) at 25°C. **(A, B, E, F)** Plant fresh weight (FW) was measured (in grams) 14 days post inoculation. **(C, D, G, H)** Disease symptoms were scored at 14 days post inoculation. Means followed by a common letter are not significantly different by Kruskal-Wallis with Dunn’s *post hoc* test and Benjamini-Hochberg correction for FW **(A, B, E, F)** or Mann-Whitney U test with Benjamini-Hochberg correction for disease symptoms **(C, D, G, H)** at the 5% level of significance. FW box plots are colored by strain type: mock/WT (white), knockouts (light grey), in locus complementation (medium grey), ectopic complementation (dark grey).

To verify that the observed phenotype is specifically due to inactivation of the targeted gene, knockout strains were complemented with the original gene. Complementation of Forc016 *ECC1a* knockout strains with *ECC1a^Forc^* restored virulence on cucumber to varying degrees (**Figures 5E–H**). *In locus* complementation fully restored wild-type levels of disease symptoms and fresh weight consistently throughout several repetitions, whereas ectopic transformation resulted in either full or partial restoration of virulence (**Supplementary Figures S9**, **S11**). In contrast, despite consistent reduction in virulence for several independent Forc016*ΔECC1b* mutants (**Figures 5B, D**), complementation with *ECC1b*, either ectopically or *in locus*, failed to restore the wild-type phenotype to Forc016*ΔECC1b-1*. We conclude that, while results are mixed for ECC1b, ECC1a^Forc^ contributes to virulence towards cucumber in Forc016.

Having established that *ECC1a^Forc^* contributes to virulence on cucumber of Forc016, we then asked whether replacing ECC1a*^Fom^* with ECC1a*^Forc^* in a Fom strain would result in gain of pathogenicity to cucumber. Complementation of Fom005*ΔECC1a-1* with *ECC1a^Forc^* did not lead to disease development (**Figures 5A–D**). This indicates that, while *ECC1a^Forc^* contributes to virulence in Forc016, its presence in the Fom005 background is not sufficient to gain pathogenicity towards cucumber. In contrast, replacing *ECC1a^Forc^* with *ECC1a^Fom^ in locus* in Forc016 significantly reduced virulence towards cucumber (**Figures 5E, G**), which corresponds with results from a previous study in which *ECC1a^Fom^* was ectopically introduced into a Forc016 background (Li et al., 2021). Together, these data show that *ECC1a^Forc^* contributes to Forc virulence towards cucumber and confirm that *ECC1a^Fom^* acts as an avirulence factor for cucumber.

### 3.5 *ECC1a* and *ECC1b* knockout strains of Forc016 and Fom005 show differential loss of virulence towards melon

**Figure 6:**
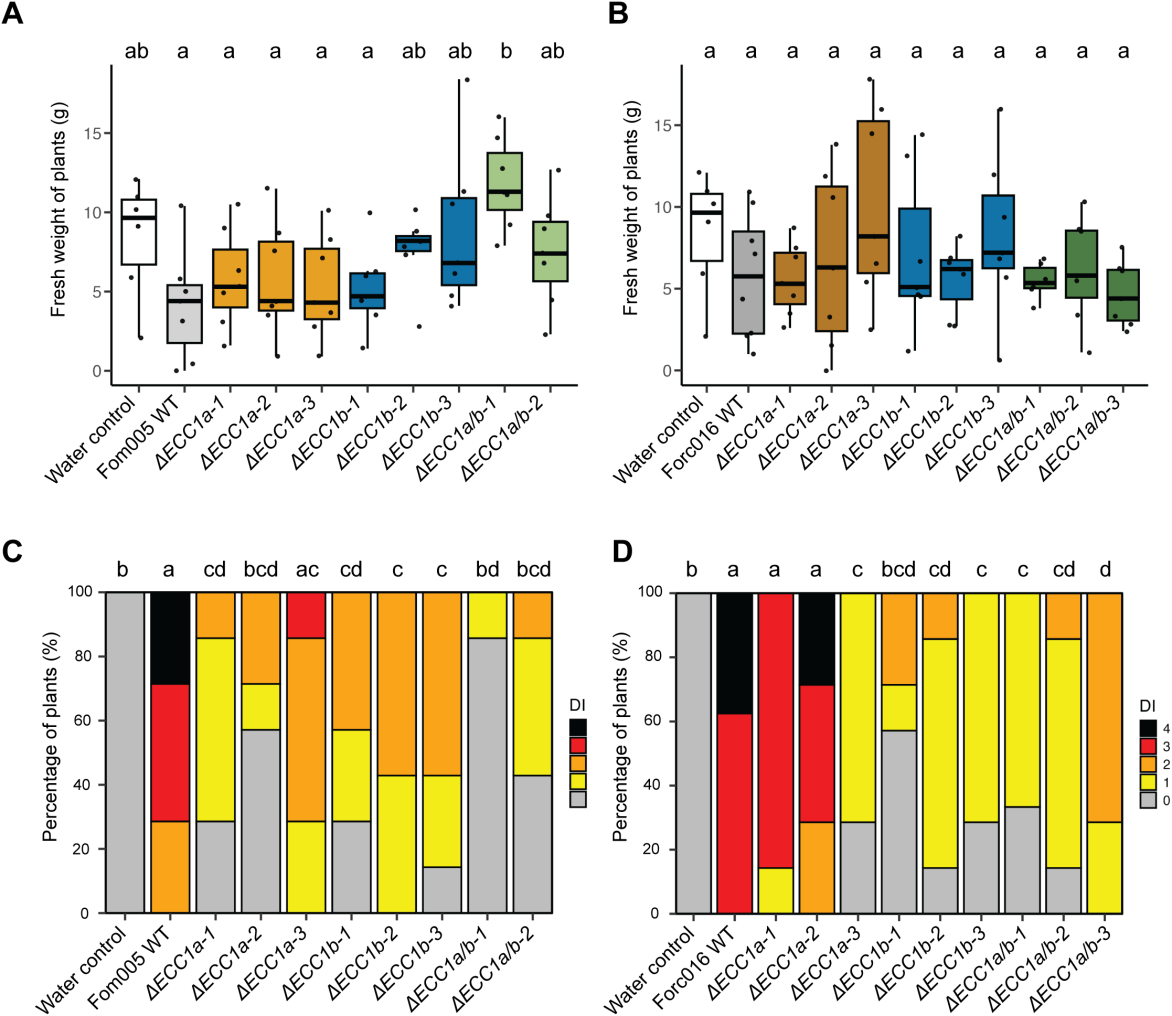
*ECC1a* and *ECC1b* knock-out strains of Forc016 and Fom005 show differential loss of virulence towards melon. Nine-day-old melon (*Cucumis melo* cv. *Cha-T*) seedlings were inoculated with water (mock), WT, *ΔECC1a*, *ΔECC1b* and *ΔECC1a/b* knockout mutants of Fom005 **(A, C)** or Forc016 **(B, D)** (n=7) at 25°C. **(A, B)** Plant fresh weight (FW) was measured (in grams) 14 days post inoculation. (**C, D**) Disease symptoms were scored 14 days post inoculation. Means followed by a common letter are not significantly different by Kruskal-Wallis with Dunn’s *post hoc* test and Benjamini-Hochberg correction for FW **(A, B)** or Mann-Whitney U test with Benjamini-Hochberg correction for disease symptoms **(C, D)** at the 5% level of significance.

Next, we investigated whether ECC1a and ECC1b contribute to disease on melon by testing severity of root rot and wilt symptoms in disease assays using single and double knockout mutants in Fom005 and Forc016. As in the cucumber infection assays, there were no significant differences in fresh weight and disease severity between melon plants inoculated with ORF deletion mutants and ORF disruption mutants. Fom005 mutants lacking *ECC1a*, or *ECC1b*, or both homologs, were significantly less virulent than the wild-type strain (**Figures 6C, D**). Most Forc016 *ECC1a* knockout strains remained virulent on melon, but Forc016 *ECC1b* knockout mutants showed reduced symptom severity compared to Forc016 wild type (**Figure 6D**). As expected, based on these results, double knockout mutants in Forc016 showed a similar phenotype as single *ECC1b* knockout mutants. Taken together, these results indicate that, except for ECC1a^Forc^, ECC1 homologs in both Forc and Fom contribute to virulence on melon.

**Figure 7:**
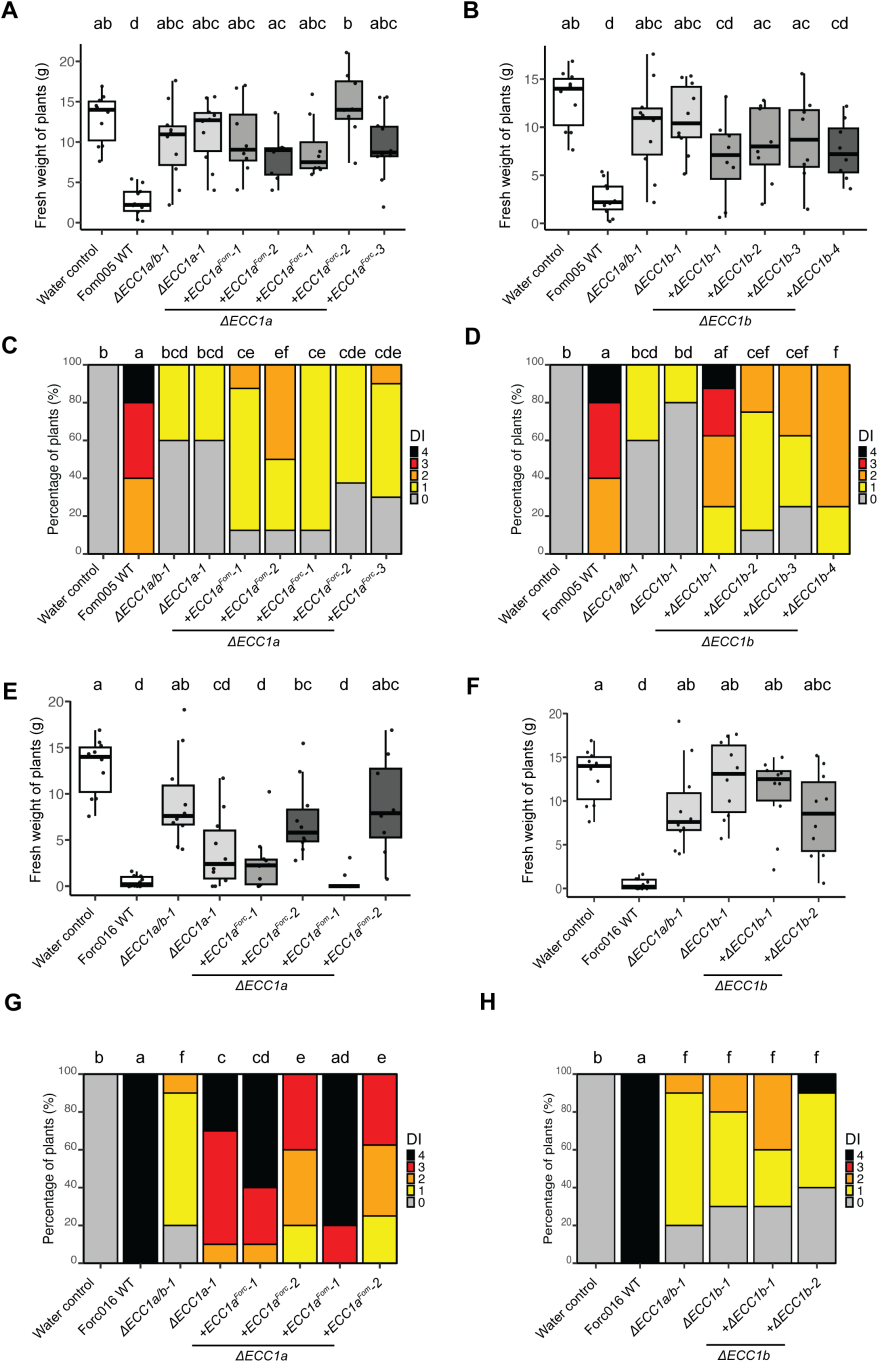
*ECC1* complementation partially restores virulence of Fom on melon and reveals contrasting roles in host specificity. Nine-day-old melon (*Cucumis melo* cv. *Cha-T*) seedlings were inoculated with water (mock), WT, *ΔECC1a*, *ΔECC1b*, *ΔECC1a/b* knockout mutants, complementation and gene replacement strains of Fom005 **(A-D)** or Forc016 **(E-H)** (n=10) at 25°C. **(A, B, E, F)** Plant fresh weight (FW) was measured (in grams) 14 days post inoculation. **(C, D, G, H)** Disease symptoms were scored at 14 days post inoculation. Means followed by a common letter are not significantly different by Kruskal-Wallis with Dunn’s *post hoc* test and Benjamini-Hochberg correction for FW **(A, B, E, F)** or Mann-Whitney U test with Benjamini-Hochberg correction for disease symptoms **(C, D, G, H)** at the 5% level of significance. FW box plots are colored by strain type: mock/WT (white), knockouts (light grey), in locus complementation (medium grey), ectopic complementation (dark grey).

To confirm that the reduced virulence of the *ECC1a* and *ECC1b* knockout mutants was due to gene deletion or disruption, we assessed whether complementation of the single knockout mutants would restore virulence (**Figure 7**). Complementation of Fom005*ΔECC1a-1* with *ECC1a^Fom^* only partially restored virulence: plant fresh weight was comparable to that of the knockout strain (**Figure 7A**), and only one strain with an ectopic insertion of *ECC1a* (Fom005*ΔECC1a-1+ECC1aFom-2*) induced more severe disease symptoms than the Fom005*ΔECC1a-1* background (**Figure 7C**). Complementation of Fom005*ΔECC1b-1* with *ECC1b^Fom^* also partially restored virulence: only the Fom005*ΔECC1b-1+ECC1b-1* strain showed full restoration of virulence (**Figures 7B, D**), and other complementation strains showed intermediate phenotypes: more severe than the knockout, but less severe than wild-type. On melon, Forc016*ΔECC1a-1* retained its virulence as observed previously (**Figure 6**), and reintroducing *ECC1a^Forc^* had no significant effect on this phenotype (**Figures 7E, G**). Although independent Forc016*ΔECC1b* mutants showed consistent loss of virulence (**Figure 6**), introducing ECC1b failed to restore the loss of virulence to wild-type phenotype to Forc016*ΔECC1b*. Therefore, the exact role of ECC1b in Forc016 infection of melon remains unclear. In contrast, both ECC1a and ECC1b appear to contribute to Fom005 virulence on melon.

*ECC1a^Fom^* and *ECC1a^Forc^* are identified as each other’s orthologs based on synteny conservation and therefore could be predicted *a priori* to have a conserved function. On the other hand, their high sequence divergence indicates functional diversification of these orthologs, e.g. with respect to a role in virulence in a specific host. To test this, *ECC1a* was replaced with *ECC1a* from the other *forma specialis*, introduced either *in locus* and/or ectopically, and virulence of these mutants on melon was assessed. Replacement of *ECC1a^Forc^* with *ECC1a^Fom^* in the Forc016*ΔECC1a-1* background yielded inconsistent results: the *in locus* replacement strain remained virulent, whereas the ectopic strain showed reduced symptom severity (**Figures 7E,G**). Conversely, replacing *ECC1a^Fom^* with *ECC1a^Forc^* in Fom did not restore virulence on melon (**Figures 7A, C**). These results indicate that *ECC1a^Fom^* is not functionally interchangeable with *ECC1a^Forc^* in melon infection and that *ECC1a^Fom^* has a *forma specialis*-specific virulence function.

### 3.6 Expression profiling of ECC1 homologs suggests a role in early infection of *Fo*

**Figure 8:**
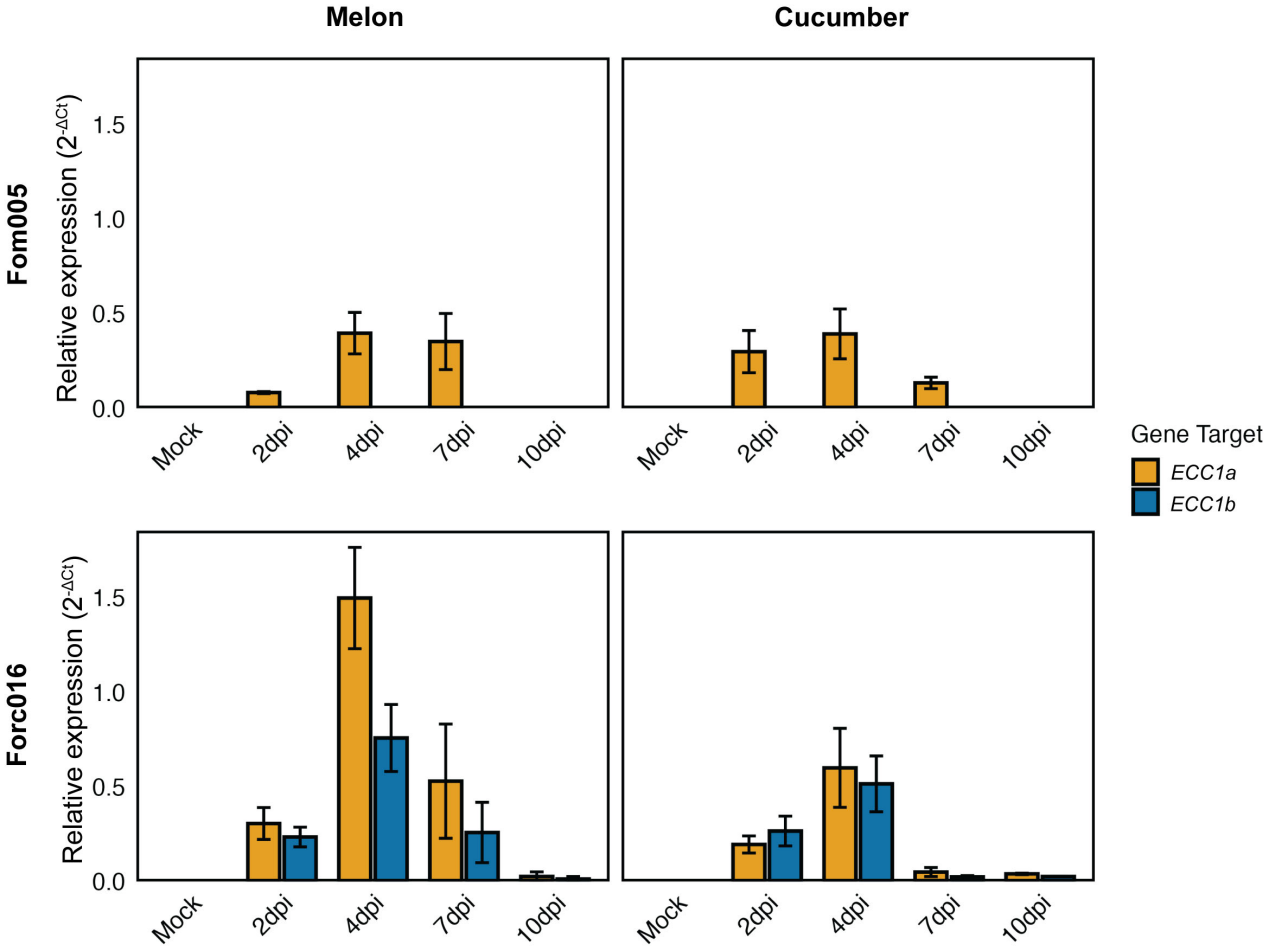
Expression profiling of *ECC1* homologs suggests a role in early infection stages of Fusarium. Seven-day-old cucumber (*Cucumis sativus* cv. *Paraiso*) and 10-day-old melon (*Cucumis melo* cv. *Cha-T*) seedlings were inoculated with wild-type Fom005 and Forc016 and sampled at 2-, 4-, 7- and 10-days post-inoculation (dpi). For each timepoint, three biological replicates were collected, each consisting of 3 pooled plants. Expression of *ECC1a* (yellow) and *ECC1b* (teal) was quantified relative to the Fusarium housekeeping gene for Translation Elongation Factor 1 alpha (*EF1α*). Error bars represent mean ± standard deviation (SD).

Having found that ECC1 homologs contribute to virulence of Fom and Forc on their respective hosts, we next investigated whether they are expressed during different stages in infection. To assess this, transcript levels of *ECC1a* and *ECC1b* relative to the Fo housekeeping gene Translation Elongation Factor 1 alpha (*EF1α*) were quantified using qPCR at 2-, 4-, 7- and 10-days post inoculation (dpi) of melon and cucumber. While the knockout and complementation assays indicated that *ECC1b* contributes to virulence of Fom005 to melon, *ECC1b* expression was not detected during melon infection. In contrast, *ECC1a^Fom^* was expressed and expression peaked at 4 dpi (**Figure 8**). As Fom005 can colonize cucumber plants, albeit without causing disease, expression of *ECC1* homologs during cucumber infection was also assessed. It was found to be comparable to that of melon infection, with similar relative expression levels at its peak at 4 dpi. This indicates that the lack of impact of ECC1a^Fom^ on virulence on cucumber is probably not due to a lack of expression.

In contrast, both *ECC1a^Forc^* and *ECC1b* were expressed during Forc016 infection of melon and cucumber. *ECC1a^Forc^* and *ECC1b* are expressed during early stages of infection: transcript levels peaked at 4 dpi and returned to very low basal levels at 10 dpi. This suggests ECC1a^Forc^ and ECC1b play a role in initial host colonization by Forc016.

## 4 Discussion

Effectors are key determinants of host specificity in Fo by promoting infection or, conversely, triggering plant immunity when recognized (Ma et al., 2010; Li et al., 2020a; Constantin et al., 2021). Fo host range is therefore thought to be shaped by the specific combination of effectors present or absent on pathogenicity chromosomes. Combined gene expression profiling and *in silico* effector prediction previously identified *ECC1a* as a candidate effector underlying host range differences between Forc and Fom. Our search for *ECC1* homologs in other Fo genome assemblies revealed that *ECC1* is part of a large gene family that has undergone multiple duplication events. This family is present in many strains that infect melon, watermelon or cucumber, while being absent in strains that do not, suggesting a role for members of this family in infection of cucurbits. To investigate their role in host-specific (a)virulence, gene knockout and replacement strategies were used, demonstrating that the *ECC1* gene family plays a role in virulence of both Forc and Fom towards cucumber and melon (**Table 1**). In addition, expression profiling indicates a potential role of *ECC1* in early infection. Together, our results reveal that both ECC1a and ECC1b are required for virulence in specific host-pathogen combinations, with functional divergence between homologs and *formae speciales*.

**Table 1:** The role of ECC1 effectors during infection of melon and cucumber.

Effectors	Melon	Cucumber
ECC1a^Fom^	Virulence	Avirulence
ECC1a^Forc^	No role	Virulence
ECC1b	Virulence	Unclear

Members of the *ECC1* family have likely been transferred on mobile pathogenicity chromosomes between strains, which complicates evolutionary reconstructions (Van Dam et al., 2017; Li et al., 2020c; van Westerhoven et al., 2025). Studying genes located on pathogenicity chromosomes present unique challenges due to the high transposon density and frequent rearrangements, deletions and duplications. This may result in underdetection of *ECC1* as duplications can be difficult to resolve in assembly of short, paired end reads, and *ECC1a* and *ECC1b* are in a 3.3 kb duplicated region. The assemblies of non-pathogenic strains and strains associated with other hosts in our dataset are all based on long reads, hence absence of *ECC1* in these strains is not likely to be due to assembly errors.

To determine how ECC1 effector homologs are involved in (a)virulence toward cucurbits, gene knockout and replacement strategies were used. To enable efficient multiplex gene editing, we employed a CRISPR/Cas9-based approach in addition to using classical Agrobacterium-mediated transformation. This was particularly important as traditional approaches to transform genes located in repeat-rich, largely heterochromatic pathogenicity chromosomes in *Fusarium* generally have low efficiency. This study is not only among the first demonstrations of multiplexed CRISPR/Cas9 targeting of *Fusarium* pathogenicity chromosomes, but this approach also allowed *in locus* complementation and replacement of *ECC1* homologs.

In this study, we confirmed earlier findings that *ECC1a^Fom^* reduces virulence of Forc016 towards cucumber (Li et al., 2021). However, disruption of *ECC1* genes in Fom005 did not result in acquiring virulence towards cucumber, nor did replacing *ECC1a^Fom^* with *ECC1a^Forc^* in Fom005. These findings indicate that the inability of Fom to cause disease in cucumber is not solely due to the recognition of ECC1a and suggests the presence of additional ‘cucumber-avirulence’ factors or the absence of factors required for cucumber infection. Host specificity in Fo is polygenic and shaped by both the presence and absence of effector genes on pathogenicity chromosomes (Ma et al., 2010; van Dam et al., 2016; Van Dam et al., 2017; Li et al., 2020a). Notably, distinct effector profiles have been identified among cucurbit-infecting isolates, supporting the idea that multiple effectors contribute to host adaptation (van Dam et al., 2016; Sabahi et al., 2021).

On melon, *ECC1* knockout mutants of Fom005 caused milder symptoms, confirming a role for ECC1a^Fom^ and ECC1b for full virulence towards this host. Given the high similarity in amino acid sequence of ECC1a^Fom^ and ECC1b, we expected some functional redundancy between these two homologs, and a larger reduction in virulence for a double knockout mutant than for the single knockout mutants. However, double knockout mutants of Fom005 did not consistently show a larger reduction in virulence on the respective host plants compared to the single knockout mutants. This may suggest compensatory mechanisms or threshold effects in virulence factor function, but could also reflect redundancy at the structural level, as suggested by the high similarity in prediction fold (**Figure 3**). Both ECC1 proteins may function together, for example as a heterodimer or by targeting the same pathway in the host. Further experiments, such as co-immunoprecipitation or protein interaction assays, could directly test these hypotheses. Complementation of the single knockout strains partially restored virulence, providing further evidence that the observed phenotypes result from loss of gene function rather than secondary effects. Although complementation was only partial, the reproducibility of these phenotypes across multiple independent deletion mutants supports a genuine role for ECC1b in virulence.

To explore whether ECC1 homologs play stage-specific roles during infection, expression dynamics were analyzed *in planta*. In Forc016, both *ECC1a* and *ECC1b* are expressed during Forc016 infection of melon and cucumber, with transcript levels peaking at 4 dpi and returning to very low basal levels at 10 dpi. Deletion of either *ECC1a* or *ECC1b* in Forc016 reduced virulence towards cucumber, suggesting that both genes are required for full virulence. In contrast, in melon, Forc016 *ECC1a* knockout mutants remained virulent, indicating that ECC1a^Forc^ is not important for melon infection. This suggests that expression does not necessarily equate to functional relevance in all host contexts. *ECC1a* expression in Fom005 peaked at 4 dpi and returned to very low basal levels at 10 dpi, a pattern similar to that observed in Forc. Such a pattern, with distinct peaks early in infection and reduced expression at later stages, resembles that observed in Fo f. sp. *lycopersici* (Fol) infecting tomato, where different effector clusters were expressed at distinct time points (Sun et al., 2022). Remarkably, *ECC1b* expression was undetectable in Fom005-infected melon and cucumber plants, despite the clear reduction in virulence upon deletion of this gene. This discrepancy may reflect technical limitations, such as the sensitivity of detection methods. Another possibility is that *ECC1b* expression is highly localized to specific infection sites or restricted to a narrow developmental window. In such cases, low-abundance transcripts may still have functional relevance, highlighting the need for higher-resolution spatiotemporal analyses. Additional approaches such as promoter-reporter fusions or *in situ* hybridization could provide higher-resolution insights into the spatiotemporal expression of *ECC1* homologs.

Reintroduction of *ECC1b* in Fom005*ΔECC1b* and Forc016*ΔECC1b* caused only partial complementation, even when the gene was reintroduced *in locus*. Similar results were obtained with a second independent knockout strain (Forc016*ΔECC1b-2*, an ORF deletion mutant instead of an ORF disruption mutant) (**Supplementary Figure S11**), making it unlikely that the phenotype is due to off-target effects of the transformation. Partial complementation has been reported in other systems, such as the basidiomycete *Ganoderma lucidum*, despite *in locus* (or *‘in situ’*) introduction (Wang et al., 2022). In our approach, a selection cassette was inserted downstream of the predicted terminator of *ECC1*, which could affect chromatin context or interfere with neighboring genes. Notably, all *ECC1* homologs are flanked by sequences that encode a protein with a necrosis inducing protein domain. These proteins are part of the Nep1-like protein (NLP) family and are involved in pathogenicity of other Fo strains (Bae et al., 2006; Gijzen and Nürnberger, 2006). Moreover, *ECC1* homologs differ in genomic context: *ECC1a* is located adjacent to a gene encoding a hAT C-terminal dimerization domain containing protein (*hATd*) (**Figure 1B**), which may influence gene expression through local chromatin remodeling (Essers et al., 2000; Rubin et al., 2001). In contrast, *ECC1b* does not flank a hAT-associated domain gene, but lies near a Rhodopsin domain-containing gene (*RHOd*) of unclear function and expression status (**Figure 1B**). Although located downstream, such neighboring elements could influence the accessibility or activity of *ECC1*.

Alternatively, the partial complementation may be due to polar effects on adjacent genes caused by the insertion of the selection cassette. Such polar effects could disrupt the expression downstream or nearby genes, including *NPP1d* or *RHOd*, which may play a role in pathogenicity. Additional expression profiling of *ECC1b* and its neighboring genes in the complementation strains could help distinguish between effects caused by local chromatin environment, insertional interference or disruption of adjacent gene function.

While ECC1a^Fom^ acts as an avirulence factor in cucumber, ECC1b, which differs only by two amino acids, does not appear to trigger recognition, despite being expressed by Forc016 during infection. This raises the possibility that minor amino acid differences may influence either host recognition or target specificity. Notably, the two amino acid substitutions are located in the putative pro-domain, upstream of a potential Kex2 protease cleavage site (**Figure 1C**). Although little is known about the precise role of pro-domains in fungi, they have been shown to contribute to protein folding, localization and activity, and have been proposed to function as intramolecular chaperones (Outram et al., 2021). Identifying host targets could help clarify whether the differences in recognition result from altered effector-host interactions, or from indirect effects on protein stability and delivery.

Our results demonstrate that the ECC1 family contributes to host-specific virulence in Fo. We show that ECC1a and ECC1b have diverged both functionally and in their expression profiles across *formae speciales* and hosts. The expression peak at 4 dpi suggests a role in early, presumably biotrophic colonization, and structural features may support an intracellular mode of action. Protein structure predictions combined with DALI analysis revealed that members of the ECC1 family share structural similarity to ToxA and known ToxA-like fungal effectors (FOXGR_015533, SIX7, SIX8 and Avr2). This structural resemblance suggests a conserved fold that may underpin a common mechanism of host interaction. Members of the ToxA-like fungal effector family, like Avr2 (Houterman et al., 2008) and SIX8 (Aalders et al., 2024), have intracellular targets, meaning that these effectors are translocated or taken up by host cells. Additionally, ECC1 lacks cysteine residues, often involved in forming intramolecular disulfide bridges that help stabilize proteins that function in extracellular spaces of the host. While this is consistent with an intracellular role, localization of ECC1 proteins remains to be established experimentally.

There is evidence that ToxA-like effectors can function in pairs, like *AVR2-SIX5* (Ma et al., 2015) and *SIX8-PSE1* (Ayukawa et al., 2021). Unlike these gene pairs, *ECC1* genes share their upstream regions with genes encoding non-secreted proteins (*4CLL7* or *RHOd*), and share their downstream regions with *NPP1d*, making functional linkage less likely.

Together, these results indicate that the ECC1 family constitutes a structurally conserved but sequence-diverse effector family with potential functional specialization across subfamilies. Moving forward, validating subcellular localization, and host targets, as well as examining promoter and chromatin context will be essential to unravel how ECC1 diversification contributes to Fo host compatibility.

## Data Availability

The original contributions presented in the study are included in the article/**Supplementary Material**. Further inquiries can be directed to the corresponding author.

## Supplementary material

Supplementary Figure 1 Phylogenic tree of Fo strains with presence/absence of ECC1 genotypes A phylogenetic tree based on a concatenated alignment of single copy conserved genes (BUSCO), in which strains are colored according to *forma specialis*. One branch (Focpep1) is shortened for visibility. On the right, each ECC1 genotype from **Figure 2** is represented as a column, where black indicates that this genotype is absent in this strain, white indicates that the genotype is present (possibly with more than one copy), and grey indicates that this strains probably has a copy, but it was disrupted due to assembly issues. ECC1 subfamilies are indicated below the different genotypes. The ECC1 family is, in this dataset, limited to strains that infect a member of the cucurbits, and is not present in all cucurbit-infecting strains. Strains that infect melon, watermelon and/or cucumber and do not have an ECC1 homolog, are indicated with red rectangles. Some genotypes, such as that of subfamily 2, 3a, 3d, 4a and 4b occur in strains that are not in the same phylogenetic clade.

Supplementary Figure 2 PCR-based verification of *in locus* integration of donor DNA. Genomic DNA was used as template to confirm correct integration of the donor construct at the target loci. Each PCR reaction included one primer located within the resistance cassette and a second primer positioned outside the homologous flank used for recombination (i.e., in genomic DNA), resulting in an amplicon only if correct *in locus* integration occurred. **(A)** PCR verification of integration at the *ECC1a* locus. **(B-D)** PCR verification of integration at the *ECC1b* locus. **(E)** Table summarizing primers and expected fragment sizes. **(F)** Table summarizing the gel lane labels and corresponding fungal strains and controls. The molecular weight marker used was FastRuler High Range (ThermoFisher Scientific), except where otherwise specified (‘M2’), in which case the 1 kb Plus DNA Ladder (ThermoFisher Scientific) was used.

Supplementary Figure 3 PCR-based confirmation of presence of *ECC1a* and *ECC1b* loci in ectopic transformants. Genomic DNA was used as a template to verify that ectopic transformants retain the *ECC1a* and *ECC1b* loci. Primers were designed to amplify a region within each target locus. **(A, B)** PCR amplification of the *ECC1a* locus. **(C, D)** PCR amplification of the *ECC1b* locus. **(E)** Table summarizing primers and expected fragment sizes. **(F)** Table summarizing gel lane labels and corresponding fungal strains and controls. The molecular weight marker used was FastRuler High Range (ThermoFisher Scientific).

Supplementary Figure 4 Disease severity index used for scoring Fusarium infection in cucumber. **(A)** Representative cucumber and **(E)** melon plants displaying the range of disease symptoms corresponding to severity scores from 0 to 4 at 14 days post inoculation: 0 indicates no symptoms; 1, slight discoloration (browning)/root rot symptoms, only at tip of main root; 2, discoloration or root rot symptoms and stem lesions visible aboveground, growth distortion; 3, very clear root rot symptoms of the entire root system, often with a large lesion extending above the cotyledons, severe growth distortion and wilting; 4, plant either dead or very small and wilted. **(B-D)** examples of plants with distinct symptom categories **(B)** slight root rot symptoms at the tip of the main root (score 1) **(C)** Root rot symptoms (score 2) **(D)** Severe stem lesions of dead plant (score 4).**(E-F)** example of a melon plant (score 3) with distinct symptom categories: severe stem lesions and root rot/browning.

Supplementary Figure 5 Representative images of cucumber seedlings inoculated with *ECC1* knockout mutants at 14dpi. Seven-day-old cucumber (*Cucumis sativus* cv. *Paraiso*) seedlings were inoculated with water (mock), WT, ΔECC1a, ΔECC1b and ΔECC1a/b knockout mutants of Fom005 or Forc016 (n=10) at 25°C. Photographs show five representative plants per treatment taken at 14 days post inoculation.

Supplementary Figure 6 Representative images of melon seedlings inoculated with ECC1 knockout mutants at 14dpi. Nine-day-old melon (*Cucumis melo* cv. *Cha-T*) seedlings were inoculated with water (mock), WT, ΔECC1a, ΔECC1b and ΔECC1a/b knockout mutants of Fom005 **(A, C)** or Forc016 **(B, D)** (n=7) at 25°C. Photographs show five representative plants per treatment taken at 14 days post inoculation.

Supplementary Figure 7 Representative images of cucumber seedlings inoculated with ECC1 knockout, complementation and gene replacement mutants at 14dpi. Seven-day-old cucumber (*Cucumis sativus* cv. *Paraiso*) seedlings were inoculated with water (mock), WT, *ΔECC1a*, *ΔECC1b* and *ΔECC1a/b* knockout mutants of Fom005 or Forc016 (n=10) at 25°C. Photographs show five representative plants per treatment taken at 14 days post inoculation.

Supplementary Figure 8 Representative images of melon seedlings inoculated with ECC1 knockout, complementation and gene replacement at 14dpi. Nine-day-old melon (*Cucumis melo* cv. *Cha-T*) seedlings were inoculated with water (mock), WT, ΔECC1a, ΔECC1b and ΔECC1a/b knockout mutants of Fom005 **(A, C)** or Forc016 **(B, D)** (n=10) at 25°C. Photographs show five representative plants per treatment taken at 14 days post inoculation.

Supplementary Figure 9 Complementation of ECC1 partially restores virulence of Forc on cucumber and reveals host-specific roles. Seven-day-old cucumber (*Cucumis sativus* cv. *Paraiso*) seedlings were inoculated with water (mock), WT, ΔECC1a, ΔECC1b, ΔECC1a/b knockout mutants, complementation and gene replacement strains of Fom005 **(A, C)** or Forc016 **(B, D)** (n=8) at 25°C. **(A, B)** Plant fresh weight (FW) was measured (in grams) 14 days post inoculation. **(C, D)** Disease symptoms were scored 14 days post inoculation. Means followed by a common letter are not significantly different by Kruskal-Wallis with Dunn’s *post hoc* test and Benjamini-Hochberg correction for FW **(A, B)** or Mann-Whitney U test with Benjamini-Hochberg correction for disease symptoms **(C, D)** at the 5% level of significance. FW box plots are colored by strain type: mock/WT (white), knockouts (light grey), in locus complementation (medium grey), ectopic complementation (dark grey).

Supplementary Figure 10 Fom005 knockout and replacement strains do not show virulence towards cucumber. Seven-day-old cucumber (*Cucumis sativus* cv. *Paraiso*) seedlings were inoculated with water (mock), WT, ΔECC1a, ΔECC1b, ΔECC1a/b knockout mutants, complementation and gene replacement strains of Fom005 (n=8) at 25°C. **(A)** Plant fresh weight (FW) was measured (in grams) 14 days post inoculation. **(B)** Disease symptoms were scored 14 days post inoculation. Means followed by a common letter are not significantly different by Kruskal-Wallis with Dunn’s *post hoc* test and Benjamini-Hochberg correction for FW **(A)** or Mann-Whitney U test with Benjamini-Hochberg correction for disease symptoms **(B)** at the 5% level of significance. FW box plots are colored by strain type: mock/WT (white), knockouts (light grey), in locus complementation (medium grey), ectopic complementation (dark grey).

Supplementary Figure 11 Complementation of ECC1 partially restores virulence of Forc on cucumber and reveals host-specific roles. Seven-day-old cucumber (*Cucumis sativus* cv. *Paraiso*) seedlings were inoculated with water (mock), WT, ΔECC1a, ΔECC1b, ΔECC1a/b knockout mutants, complementation and gene replacement strains of Forc016 (n=9) at 25°C. **(A)** Plant fresh weight (FW) was measured (in grams) 14 days post inoculation. **(B)** Disease symptoms were scored 14 days post inoculation. Means followed by a common letter are not significantly different by Kruskal-Wallis with Dunn’s *post hoc* test and Benjamini-Hochberg correction for FW **(A)** or Mann-Whitney U test with Benjamini-Hochberg correction for disease symptoms **(B)** at the 5% level of significance

Supplementary Figure 12
*ECC1* complementation partially restores virulence of Fom on melon and reveals contrasting roles in host specificity. Nine-day-old melon (*Cucumis melo* cv. *Cha-T*) seedlings were inoculated with water (mock), WT, ΔECC1a, ΔECC1b, ΔECC1a/b knockout mutants, complementation and gene replacement strains of Fom005 **(A, C)** or Forc016 **(B, D)** (n=5) at 25°C. **(A, B)** Plant fresh weight (FW) was measured (in grams) 14 days post inoculation. **(C, D)** Disease symptoms were scored 14 days post inoculation. Means followed by a common letter are not significantly different by Kruskal-Wallis with Dunn’s *post hoc* test and Benjamini-Hochberg correction for FW **(A, B)** or Mann-Whitney U test with Benjamini-Hochberg correction for disease symptoms **(C, D)** at the 5% level of significance. FW box plots are colored by strain type: mock/WT (white), knockouts (light grey), in locus complementation (medium grey), ectopic complementation (dark grey).

Supplementary Figure 13
*ECC1* complementation partially restores virulence of Fom on melon and reveals contrasting roles in host specificity. Nine-day-old melon (*Cucumis melo* cv. *Cha-T*) seedlings were inoculated with water (mock), WT, ΔECC1a, ΔECC1b, ΔECC1a/b knockout mutants, complementation and gene replacement strains of Fom005 (n=8) at 25°C. **(A)** Plant fresh weight (FW) was measured (in grams) 14 days post inoculation. **(B)** Disease symptoms were scored 14 days post inoculation. Means followed by a common letter are not significantly different by Kruskal-Wallis with Dunn’s *post hoc* test and Benjamini-Hochberg correction for FW **(A)** or Mann-Whitney U test with Benjamini-Hochberg correction for disease symptoms **(B)** at the 5% level of significance. FW box plots are colored by strain type: mock/WT (white), knockouts (light grey), in locus complementation (medium grey), ectopic complementation (dark grey).

Supplementary Figure 14 ECC1a^Forc^ does not contribute to Forc virulence on melon. Nine-day-old melon (*Cucumis melo* cv. *Cha-T*) seedlings were inoculated with water (mock), WT, ΔECC1a, ΔECC1b, ΔECC1a/b knockout mutants, complementation and gene replacement strains of Forc016 (n=8) at 25°C. **(A)** Plant fresh weight (FW) was measured (in grams) 14 days post inoculation. **(B)** Disease symptoms were scored 14 days post inoculation. Means followed by a common letter are not significantly different by Kruskal-Wallis with Dunn’s *post hoc* test and Benjamini-Hochberg correction for FW **(A)** or Mann-Whitney U test with Benjamini-Hochberg correction for disease symptoms **(B)** at the 5% level of significance. FW box plots are colored by strain type: mock/WT (white), knockouts (light grey), in locus complementation (medium grey), ectopic complementation (dark grey).

Supplementary Table 1 Dataset of public Fo genome assemblies used in this study.

Supplementary Table 2 List of PCR primers used in this study

Supplementary Table 3 Guide RNA (gRNA) oligonucleotides used for CRISPR/Cas9-mediated genome editing in Fo.

Supplementary Table 4 Fo strains used or generated in this study.

Supplementary Table 5 Primers and probes used for TaqMan-based quantitative PCR.
