# Supplementary material for: Divergent ECC1 effector homologs modulate host-specific virulence in cucurbit-infecting Fusarium oxysporum
Source: Front Cell Infect Microbiol. 2025 Sep 8;15:1656785. doi: 10.3389/fcimb.2025.1656785 (PMC12450888; doi:10.3389/fcimb.2025.1656785)
Supplement: Supplementary Figure 1 — Phylogenic tree of Fo strains with presence/absence of ECC1 genotypes A phylogenetic tree based on a concatenated alignment of single copy conserved genes (BUSCO), in which strains are colored according to forma specialis. One branch (Focpep1) is shortened for visibility. On the right, each ECC1 genotype from Figure 2 is represented as a column, where black indicates that this genotype is absent in this strain, white indicates that the genotype is present (possibly with more than one copy), and grey indicates that this strains probably has a copy, but it was disrupted due to assembly issues. ECC1 subfamilies are indicated below the different genotypes. The ECC1 family is, in this dataset, limited to strains that infect a member of the cucurbits, and is not present in all cucurbit-infecting strains. Strains that infect melon, watermelon and/or cucumber and do not have an ECC1 homolog, are indicated with red rectangles. Some genotypes, such as that of subfamily 2, 3a, 3d, 4a and 4b occur in strains that are not in the same phylogenetic clade. [file Image1.pdf]

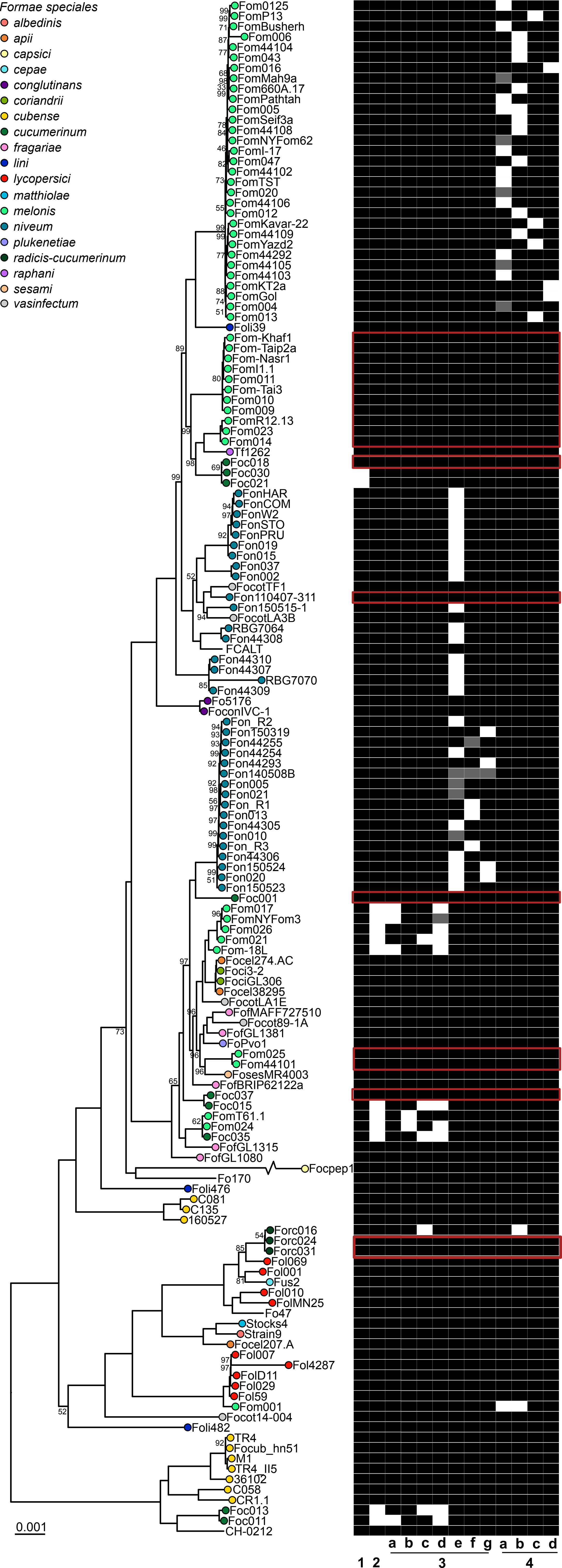

Formae speciales
albedinis
apii
capsici
cepae
conglutinans
coriandrii
cubense
cucumerinum
fragariae
lini
lycopersici
matthiolae
melonis
niveum
plukenetiae
radicis-cucumerinum
raphani
sesami
vasinfectum
Fom0125
FomP13
FomBusherh
Fom006
Fom44104
Fom043
Fom016
FomMah9a
Fom660A.17
FomPathtah
Fom005
FomSeif3a
Fom44108
FomNYFom62
FomI-17
Fom047
Fom44102
FomTST
Fom020
Fom44106
Fom012
FomKavar-22
Fom44109
FomYazd2
Fom44292
Fom44105
Fom44103
FomKT2a
FomGol
Fom004
Fom013
Foli39
Fom-Khaf1
Fom-Taip2a
Fom-Nasr1
FomI1.1
Fom011
Fom-Tai3
Fom010
Fom009
FomR12.13
Fom023
Fom014
Tf1262
Foc018
Foc030
Foc021
FonHAR
FonCOM
FonW2
FonSTO
FonPRU
Fon019
Fon015
Fon037
Fon002
FocotTF1
Fon110407-311
Fon150515-1
FocotLA3B
RBG7064
Fon44308
FCALT
Fon44310
Fon44307
RBG7070
Fon44309
Fo5176
FoconIVC-1
Fon_R2
Fon150319
Fon44255
Fon44254
Fon44293
Fon140508B
Fon005
Fon021
Fon_R1
Fon013
Fon44305
Fon010
Fon_R3
Fon44306
Fon150524
Fon020
Fon150523
Foc001
Fom017
FomNYFom3
Fom026
Fom021
Fom-18L
Focel274.AC
Foci3-2
FociGL306
Focel38295
FocotLA1E
FofMAFF727510
Focot89-1A
FofGL1381
FoPvo1
Fom025
Fom44101
FosesMR4003
FofBRIP62122a
Foc037
Foc015
FomT61.1
Fom024
Foc035
FofGL1315
FofGL1080
Focpep1
Fo170
Foli476
C081
C135
160527
Forc016
Forc024
Forc031
Fol069
Fol001
Fus2
Fol010
FolMN25
Fo47
Stocks4
Strain9
Focel207.A
Fol007
Fol4287
FolD11
Fol029
Fol59
Fom001
Focot14-004
Foli482
TR4
Focub_hn51
M1
TR4_II5
36102
C058
CR1.1
Foc013
Foc011
CH-0212
0.001
a b c d e f g a b c d
1 2 3 4
