# Supplementary figures and images for: Divergent ECC1 effector homologs modulate host-specific virulence in cucurbit-infecting Fusarium oxysporum
Source: Front Cell Infect Microbiol. 2025 Sep 8;15:1656785. doi: 10.3389/fcimb.2025.1656785 (PMC12450888; doi:10.3389/fcimb.2025.1656785)

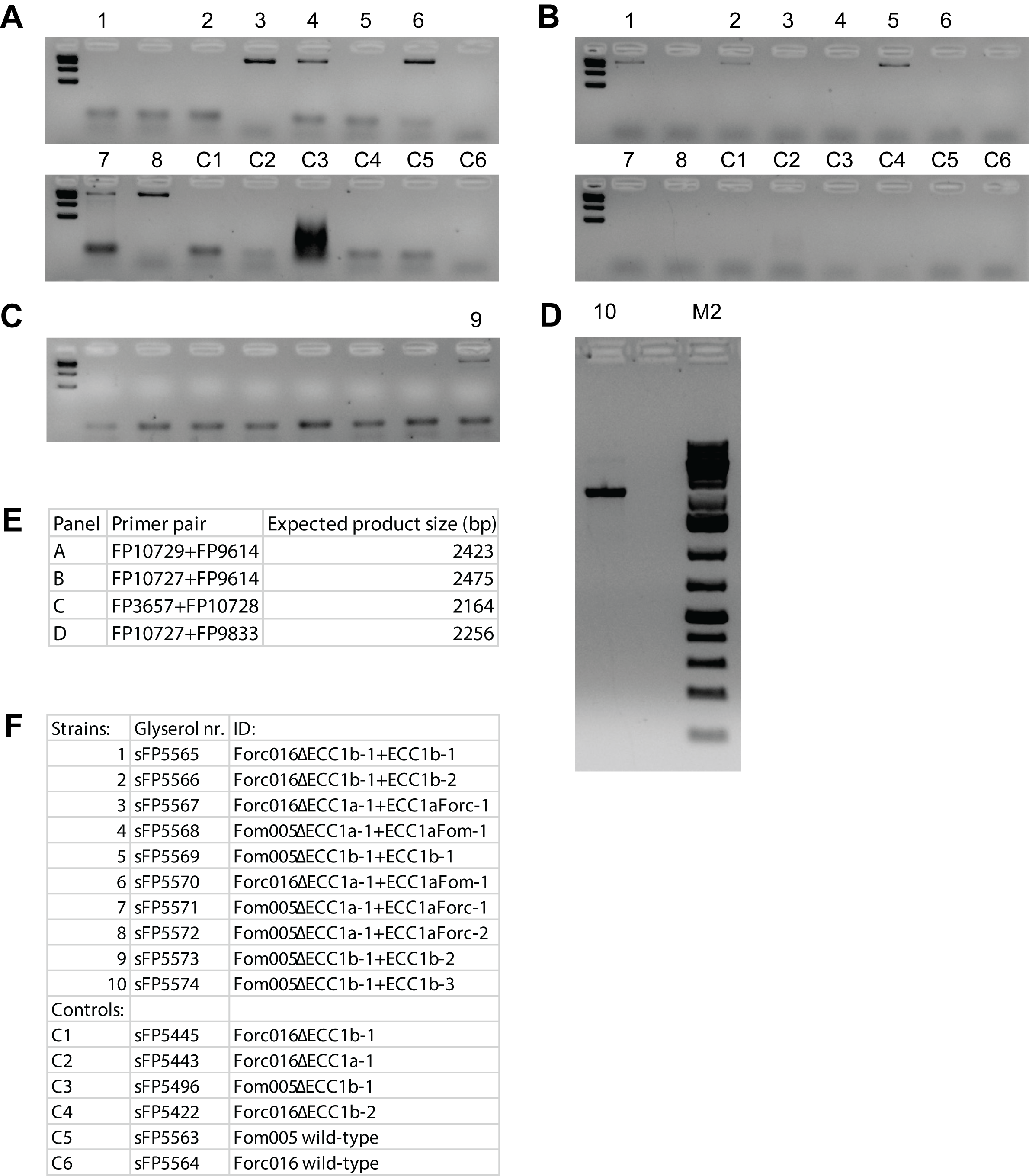

Supplement: Supplementary Figure 2 — PCR-based verification of in locus integration of donor DNA. Genomic DNA was used as template to confirm correct integration of the donor construct at the target loci. Each PCR reaction included one primer located within the resistance cassette and a second primer positioned outside the homologous flank used for recombination (i.e., in genomic DNA), resulting in an amplicon only if correct in locus integration occurred. (A) PCR verification of integration at the ECC1a locus. (B-D) PCR verification of integration at the ECC1b locus. (E) Table summarizing primers and expected fragment sizes. (F) Table summarizing the gel lane labels and corresponding fungal strains and controls. The molecular weight marker used was FastRuler High Range (ThermoFisher Scientific), except where otherwise specified (‘M2’), in which case the 1 kb Plus DNA Ladder (ThermoFisher Scientific) was used. [file Image2.tif]

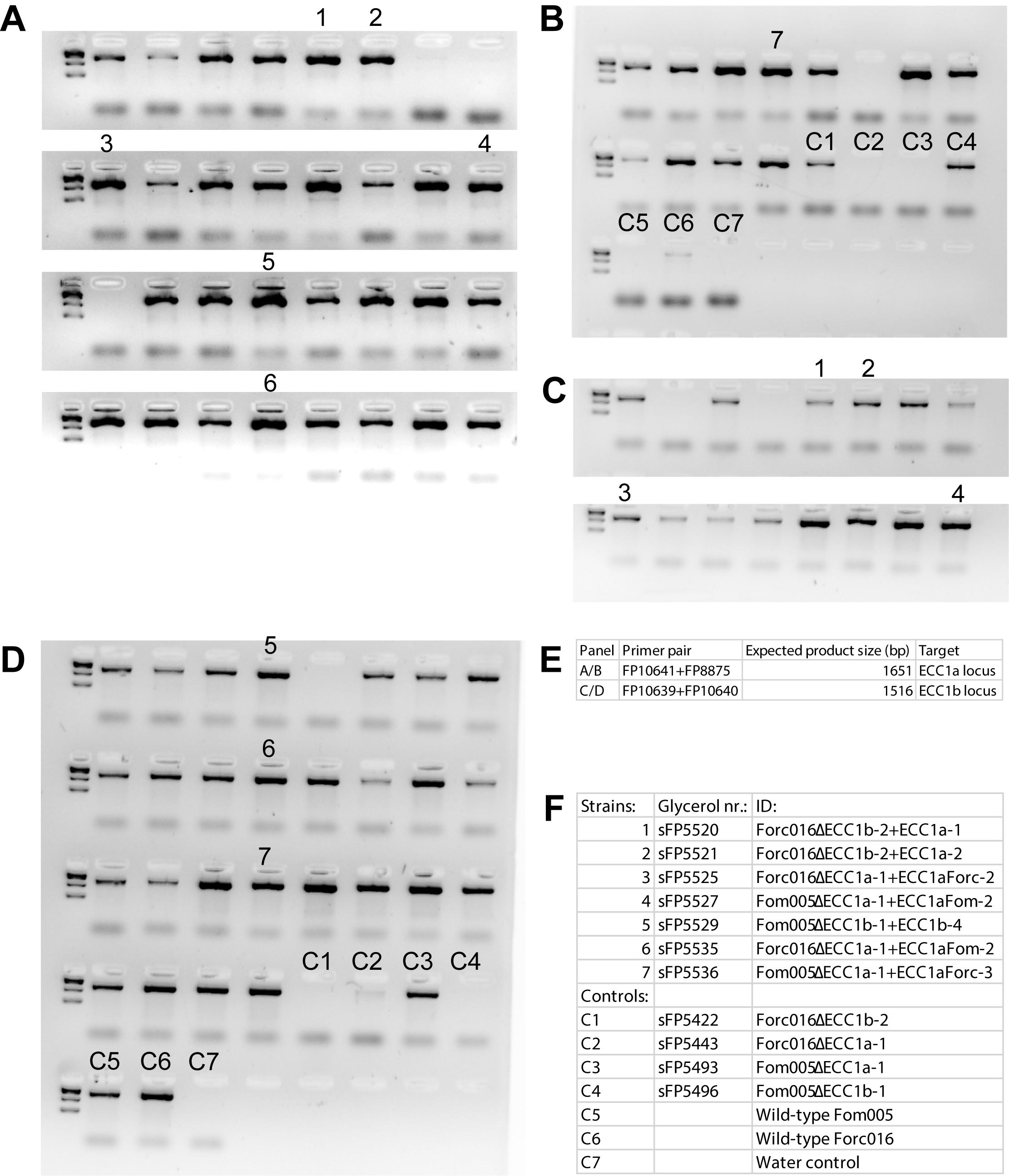

Supplement: Supplementary Figure 3 — PCR-based confirmation of presence of ECC1a and ECC1b loci in ectopic transformants. Genomic DNA was used as a template to verify that ectopic transformants retain the ECC1a and ECC1b loci. Primers were designed to amplify a region within each target locus. (A, B) PCR amplification of the ECC1a locus. (C, D) PCR amplification of the ECC1b locus. (E) Table summarizing primers and expected fragment sizes. (F) Table summarizing gel lane labels and corresponding fungal strains and controls. The molecular weight marker used was FastRuler High Range (ThermoFisher Scientific). [file Image3.tif]

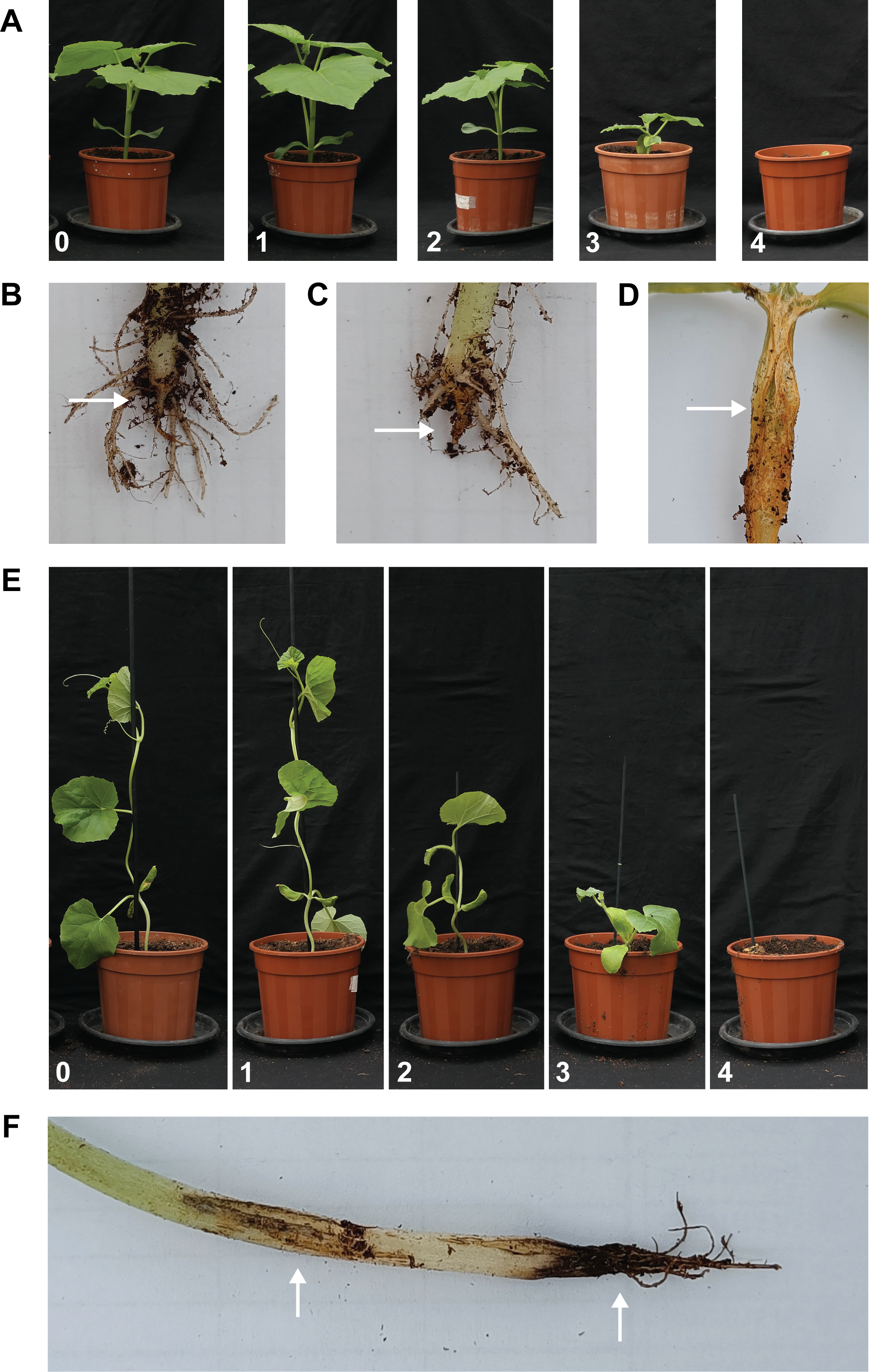

Supplement: Supplementary Figure 4 — Disease severity index used for scoring Fusarium infection in cucumber. (A) Representative cucumber and (E) melon plants displaying the range of disease symptoms corresponding to severity scores from 0 to 4 at 14 days post inoculation: 0 indicates no symptoms; 1, slight discoloration (browning)/root rot symptoms, only at tip of main root; 2, discoloration or root rot symptoms and stem lesions visible aboveground, growth distortion; 3, very clear root rot symptoms of the entire root system, often with a large lesion extending above the cotyledons, severe growth distortion and wilting; 4, plant either dead or very small and wilted. (B-D) examples of plants with distinct symptom categories (B) slight root rot symptoms at the tip of the main root (score 1) (C) Root rot symptoms (score 2) (D) Severe stem lesions of dead plant (score 4).(E-F) example of a melon plant (score 3) with distinct symptom categories: severe stem lesions and root rot/browning. [file Image4.tif]

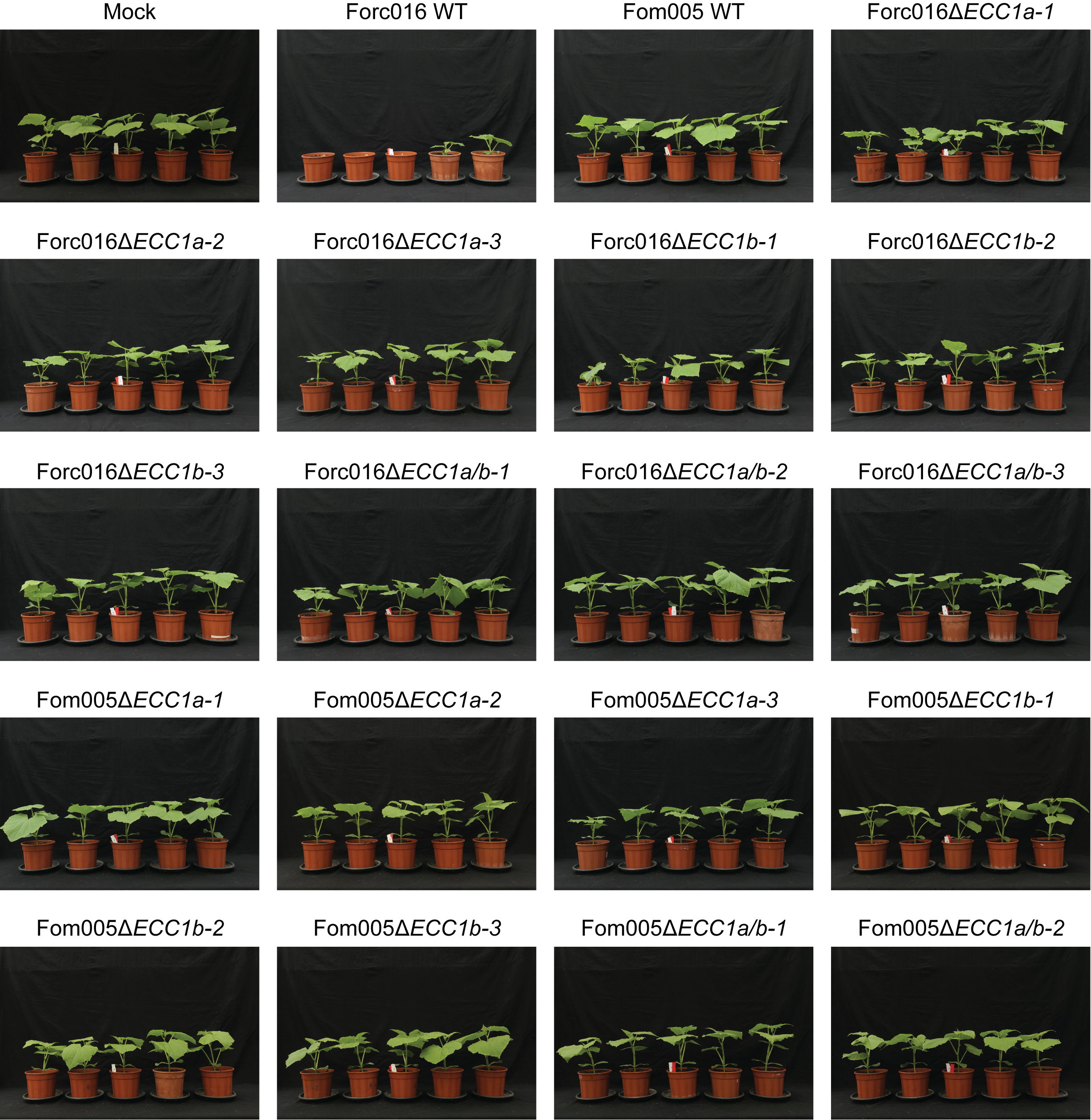

Supplement: Supplementary Figure 5 — Representative images of cucumber seedlings inoculated with ECC1 knockout mutants at 14dpi. Seven-day-old cucumber (Cucumis sativus cv. Paraiso) seedlings were inoculated with water (mock), WT, ΔECC1a, ΔECC1b and ΔECC1a/b knockout mutants of Fom005 or Forc016 (n=10) at 25°C. Photographs show five representative plants per treatment taken at 14 days post inoculation. [file Image5.tif]

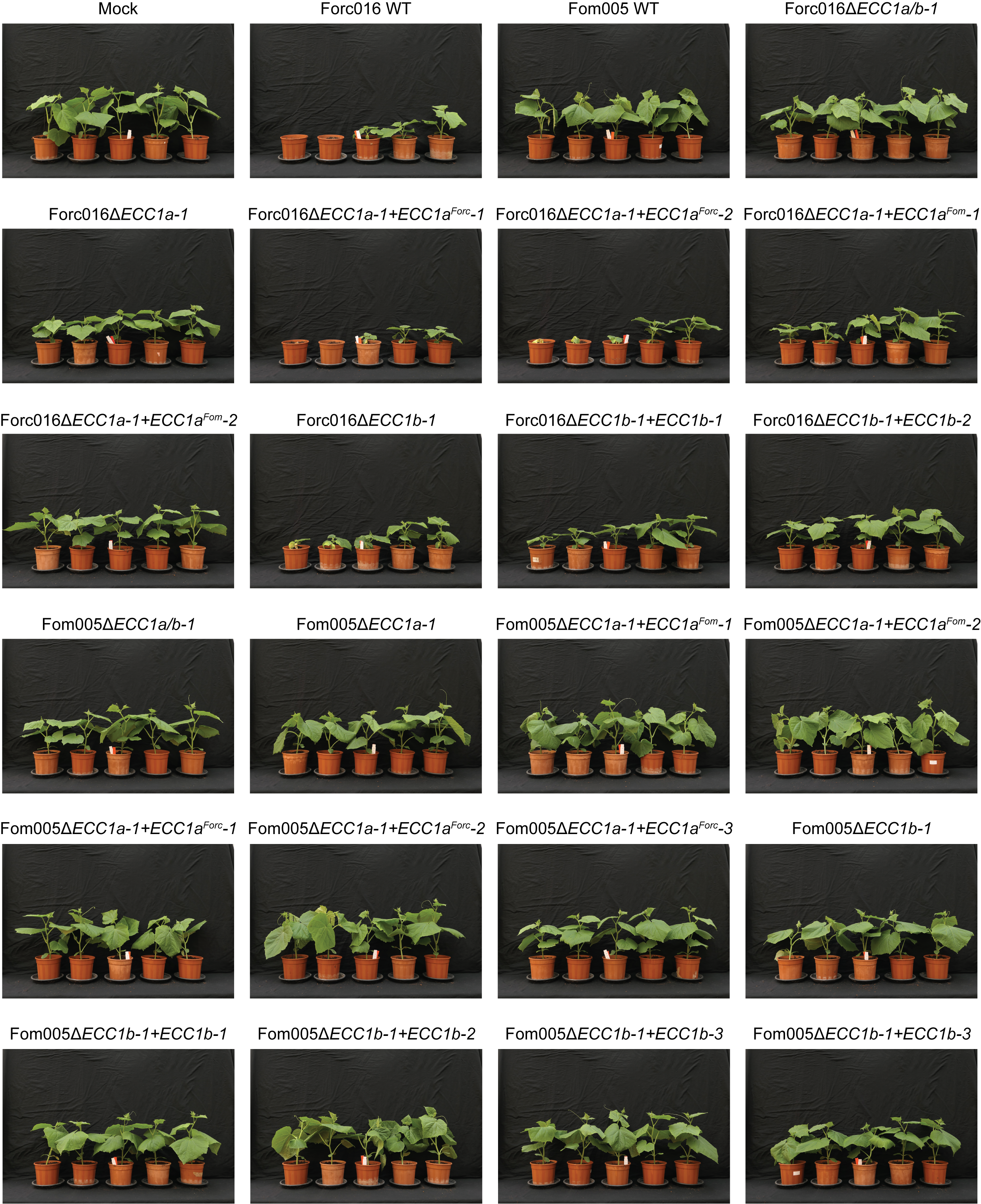

Supplement: Supplementary Figure 6 — Representative images of melon seedlings inoculated with ECC1 knockout mutants at 14dpi. Nine-day-old melon (Cucumis melo cv. Cha-T) seedlings were inoculated with water (mock), WT, ΔECC1a, ΔECC1b and ΔECC1a/b knockout mutants of Fom005 (A, C) or Forc016 (B, D) (n=7) at 25°C. Photographs show five representative plants per treatment taken at 14 days post inoculation. [file Image6.tif]

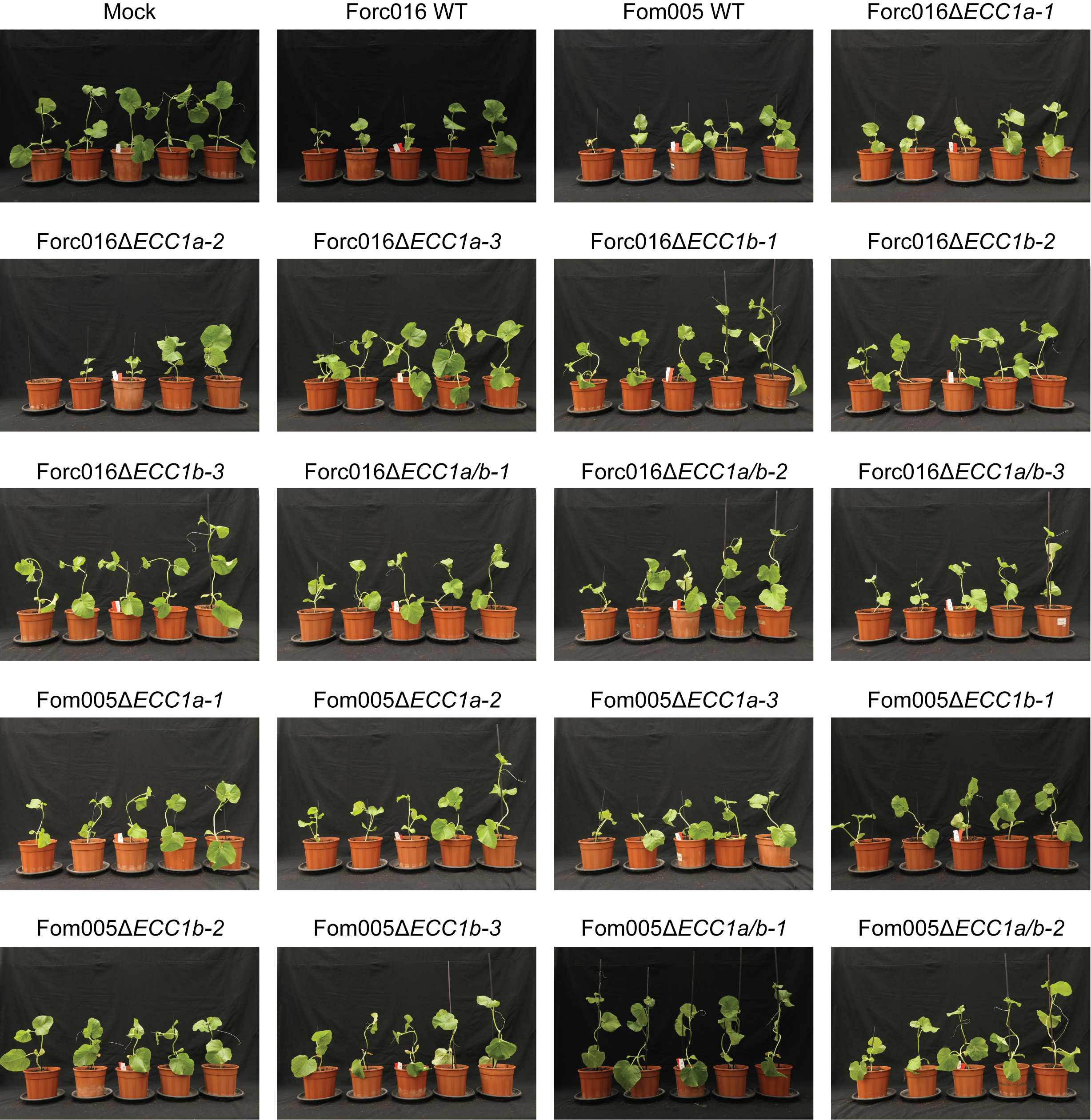

Supplement: Supplementary Figure 7 — Representative images of cucumber seedlings inoculated with ECC1 knockout, complementation and gene replacement mutants at 14dpi. Seven-day-old cucumber (Cucumis sativus cv. Paraiso) seedlings were inoculated with water (mock), WT, ΔECC1a, ΔECC1b and ΔECC1a/b knockout mutants of Fom005 or Forc016 (n=10) at 25°C. Photographs show five representative plants per treatment taken at 14 days post inoculation. [file Image7.tif]

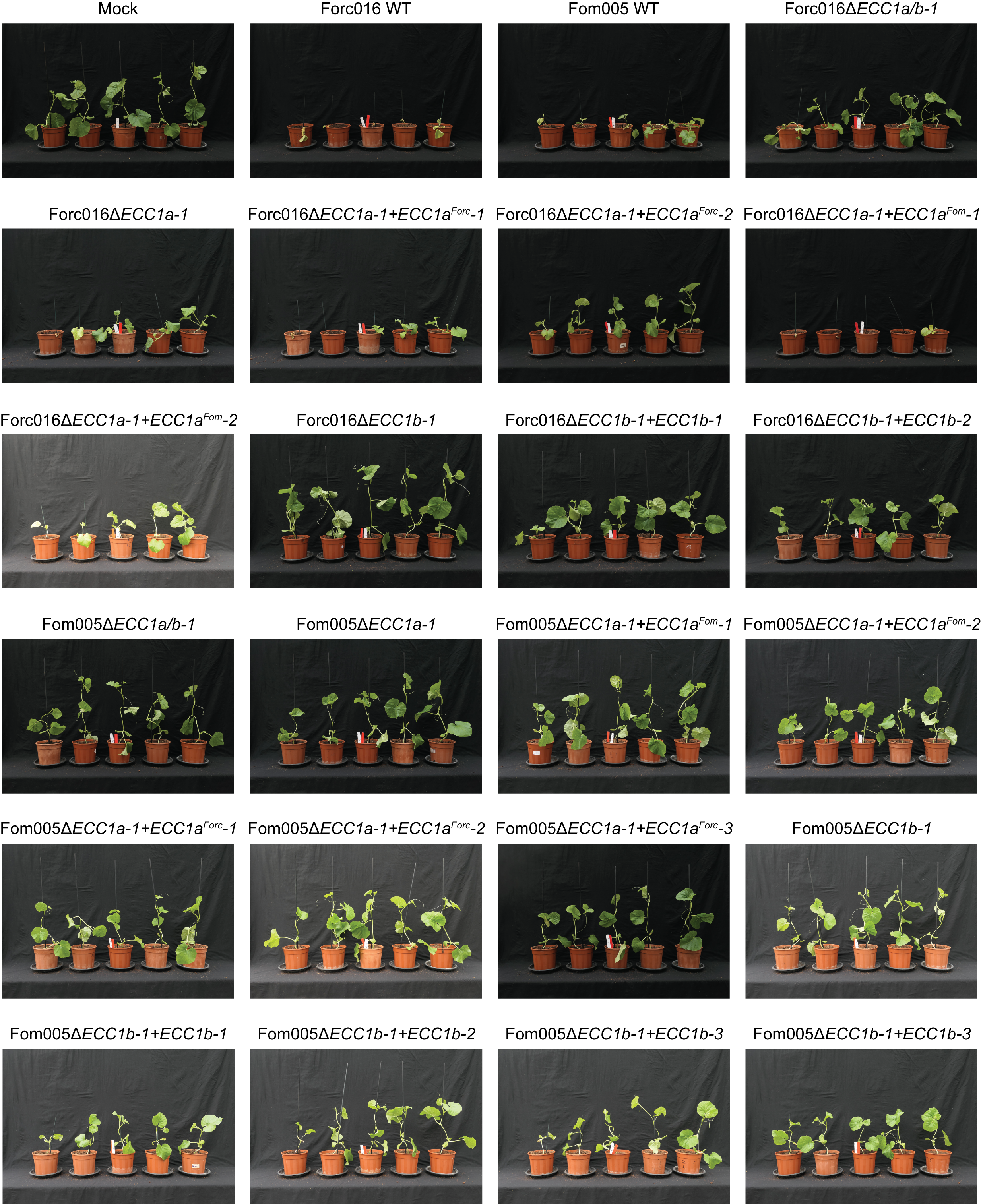

Supplement: Supplementary Figure 8 — Representative images of melon seedlings inoculated with ECC1 knockout, complementation and gene replacement at 14dpi. Nine-day-old melon (Cucumis melo cv. Cha-T) seedlings were inoculated with water (mock), WT, ΔECC1a, ΔECC1b and ΔECC1a/b knockout mutants of Fom005 (A, C) or Forc016 (B, D) (n=10) at 25°C. Photographs show five representative plants per treatment taken at 14 days post inoculation. [file Image8.tif]

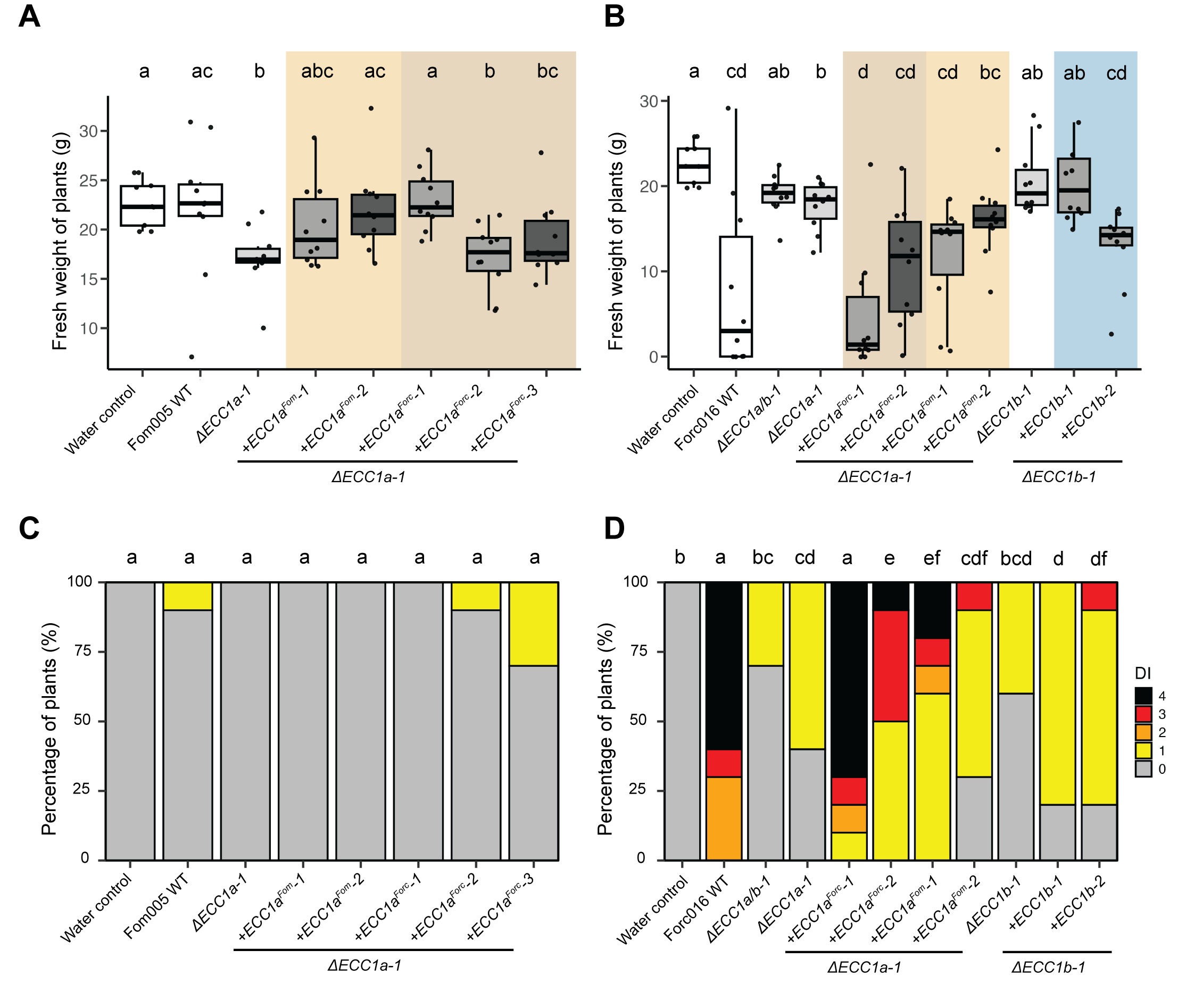

Supplement: Supplementary Figure 9 — Complementation of ECC1 partially restores virulence of Forc on cucumber and reveals host-specific roles. Seven-day-old cucumber (Cucumis sativus cv. Paraiso) seedlings were inoculated with water (mock), WT, ΔECC1a, ΔECC1b, ΔECC1a/b knockout mutants, complementation and gene replacement strains of Fom005 (A, C) or Forc016 (B, D) (n=8) at 25°C. (A, B) Plant fresh weight (FW) was measured (in grams) 14 days post inoculation. (C, D) Disease symptoms were scored 14 days post inoculation. Means followed by a common letter are not significantly different by Kruskal-Wallis with Dunn’s post hoc test and Benjamini-Hochberg correction for FW (A, B) or Mann-Whitney U test with Benjamini-Hochberg correction for disease symptoms (C, D) at the 5% level of significance. FW box plots are colored by strain type: mock/WT (white), knockouts (light grey), in locus complementation (medium grey), ectopic complementation (dark grey). [file Image9.tif]

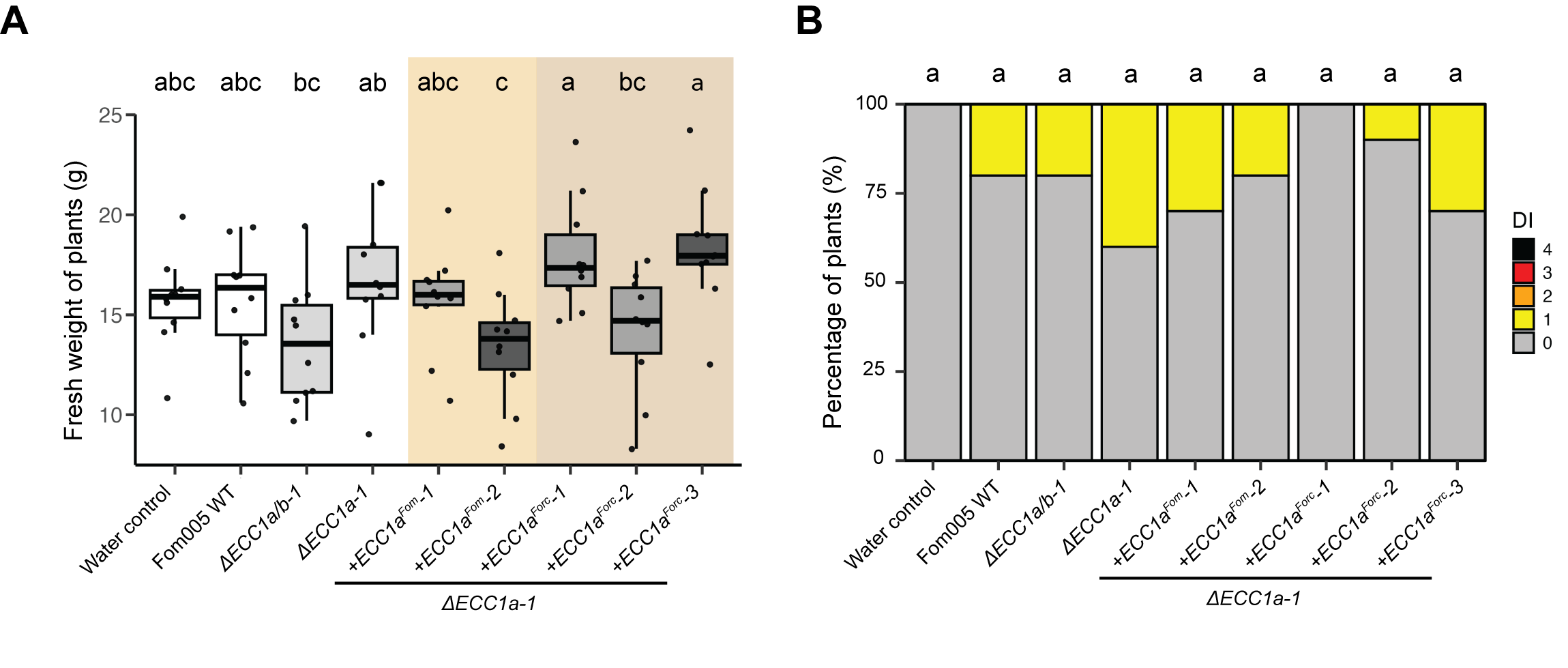

Supplement: Supplementary Figure 10 — Fom005 knockout and replacement strains do not show virulence towards cucumber. Seven-day-old cucumber (Cucumis sativus cv. Paraiso) seedlings were inoculated with water (mock), WT, ΔECC1a, ΔECC1b, ΔECC1a/b knockout mutants, complementation and gene replacement strains of Fom005 (n=8) at 25°C. (A) Plant fresh weight (FW) was measured (in grams) 14 days post inoculation. (B) Disease symptoms were scored 14 days post inoculation. Means followed by a common letter are not significantly different by Kruskal-Wallis with Dunn’s post hoc test and Benjamini-Hochberg correction for FW (A) or Mann-Whitney U test with Benjamini-Hochberg correction for disease symptoms (B) at the 5% level of significance. FW box plots are colored by strain type: mock/WT (white), knockouts (light grey), in locus complementation (medium grey), ectopic complementation (dark grey). [file Image10.tif]

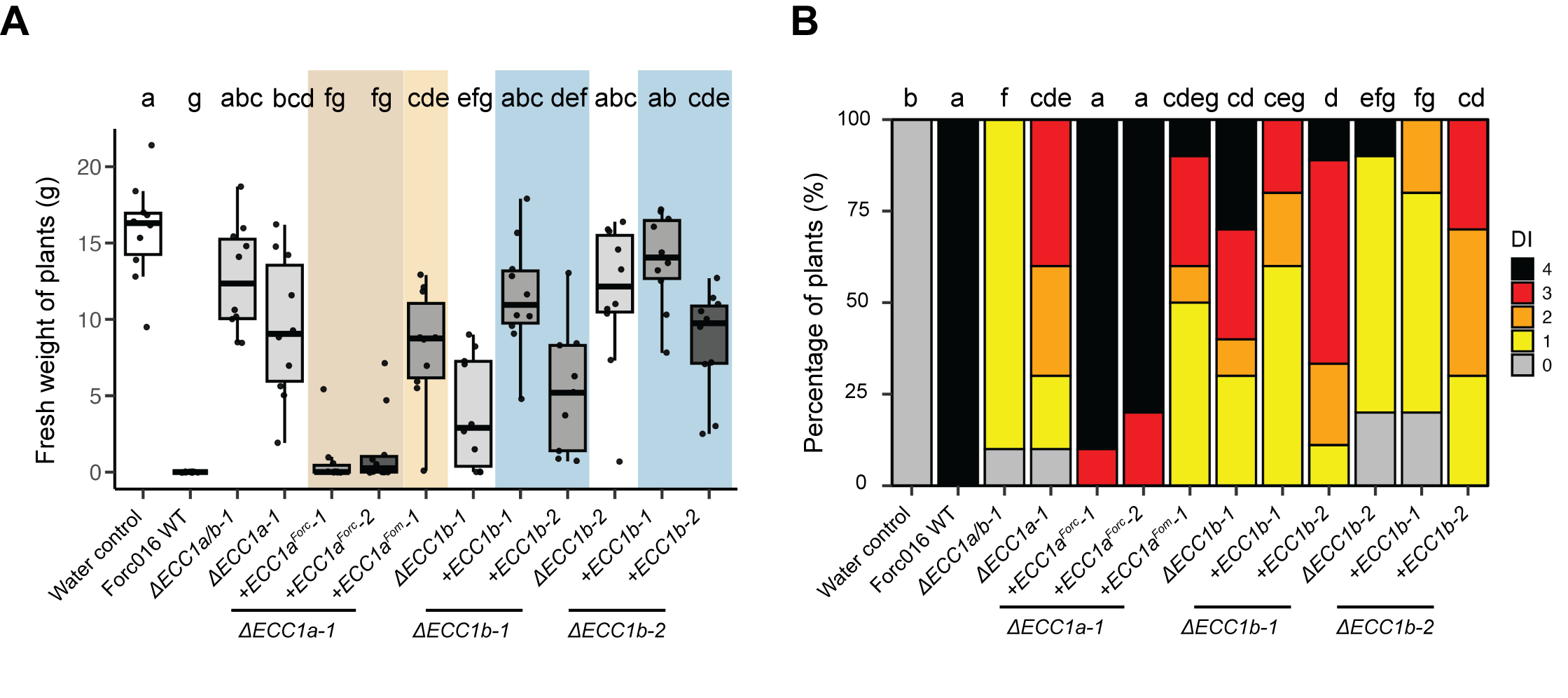

Supplement: Supplementary Figure 11 — Complementation of ECC1 partially restores virulence of Forc on cucumber and reveals host-specific roles. Seven-day-old cucumber (Cucumis sativus cv. Paraiso) seedlings were inoculated with water (mock), WT, ΔECC1a, ΔECC1b, ΔECC1a/b knockout mutants, complementation and gene replacement strains of Forc016 (n=9) at 25°C. (A) Plant fresh weight (FW) was measured (in grams) 14 days post inoculation. (B) Disease symptoms were scored 14 days post inoculation. Means followed by a common letter are not significantly different by Kruskal-Wallis with Dunn’s post hoc test and Benjamini-Hochberg correction for FW (A) or Mann-Whitney U test with Benjamini-Hochberg correction for disease symptoms (B) at the 5% level of significance [file Image11.tif]

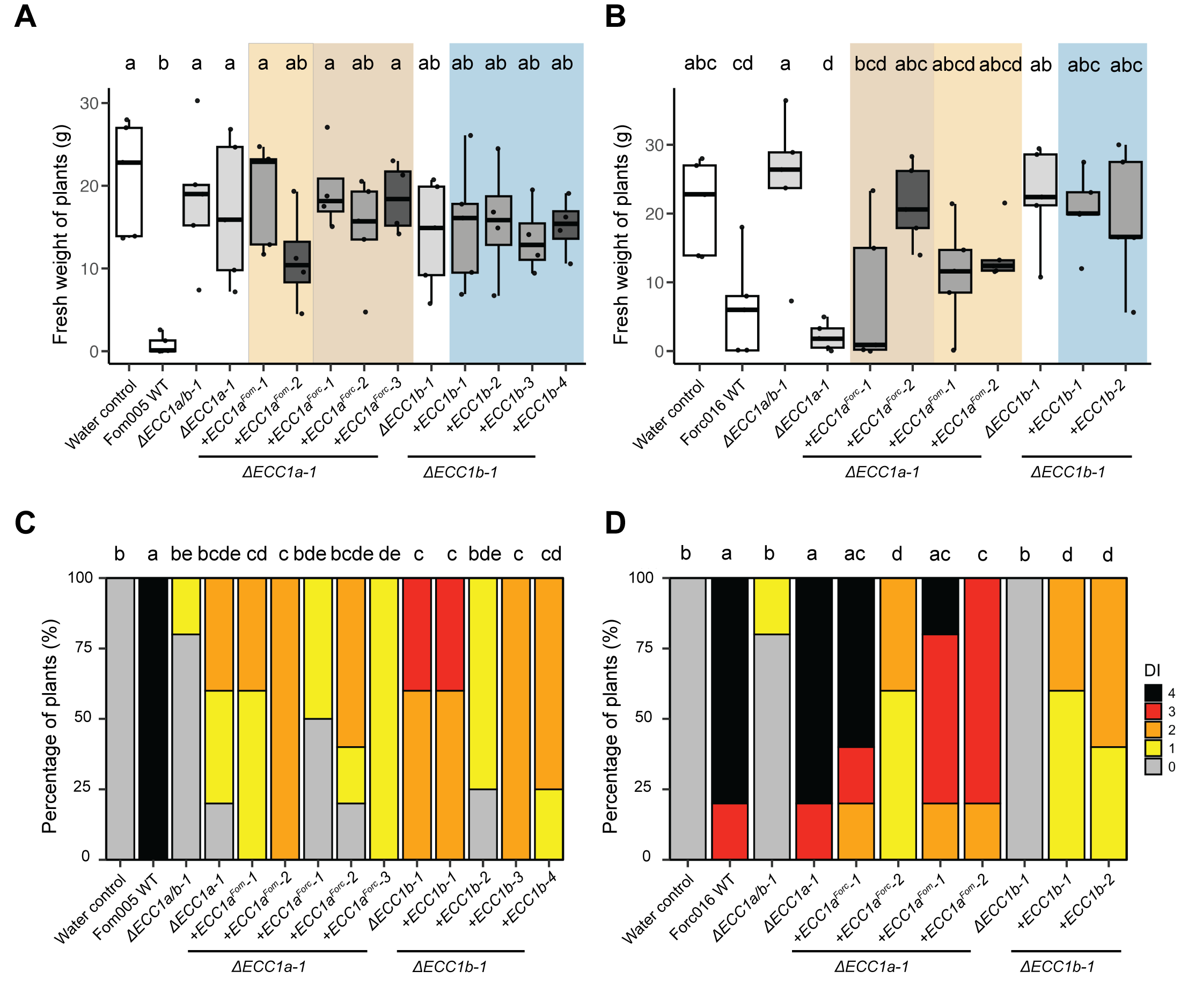

Supplement: Supplementary Figure 12 — ECC1 complementation partially restores virulence of Fom on melon and reveals contrasting roles in host specificity. Nine-day-old melon (Cucumis melo cv. Cha-T) seedlings were inoculated with water (mock), WT, ΔECC1a, ΔECC1b, ΔECC1a/b knockout mutants, complementation and gene replacement strains of Fom005 (A, C) or Forc016 (B, D) (n=5) at 25°C. (A, B) Plant fresh weight (FW) was measured (in grams) 14 days post inoculation. (C, D) Disease symptoms were scored 14 days post inoculation. Means followed by a common letter are not significantly different by Kruskal-Wallis with Dunn’s post hoc test and Benjamini-Hochberg correction for FW (A, B) or Mann-Whitney U test with Benjamini-Hochberg correction for disease symptoms (C, D) at the 5% level of significance. FW box plots are colored by strain type: mock/WT (white), knockouts (light grey), in locus complementation (medium grey), ectopic complementation (dark grey). [file Image12.tif]

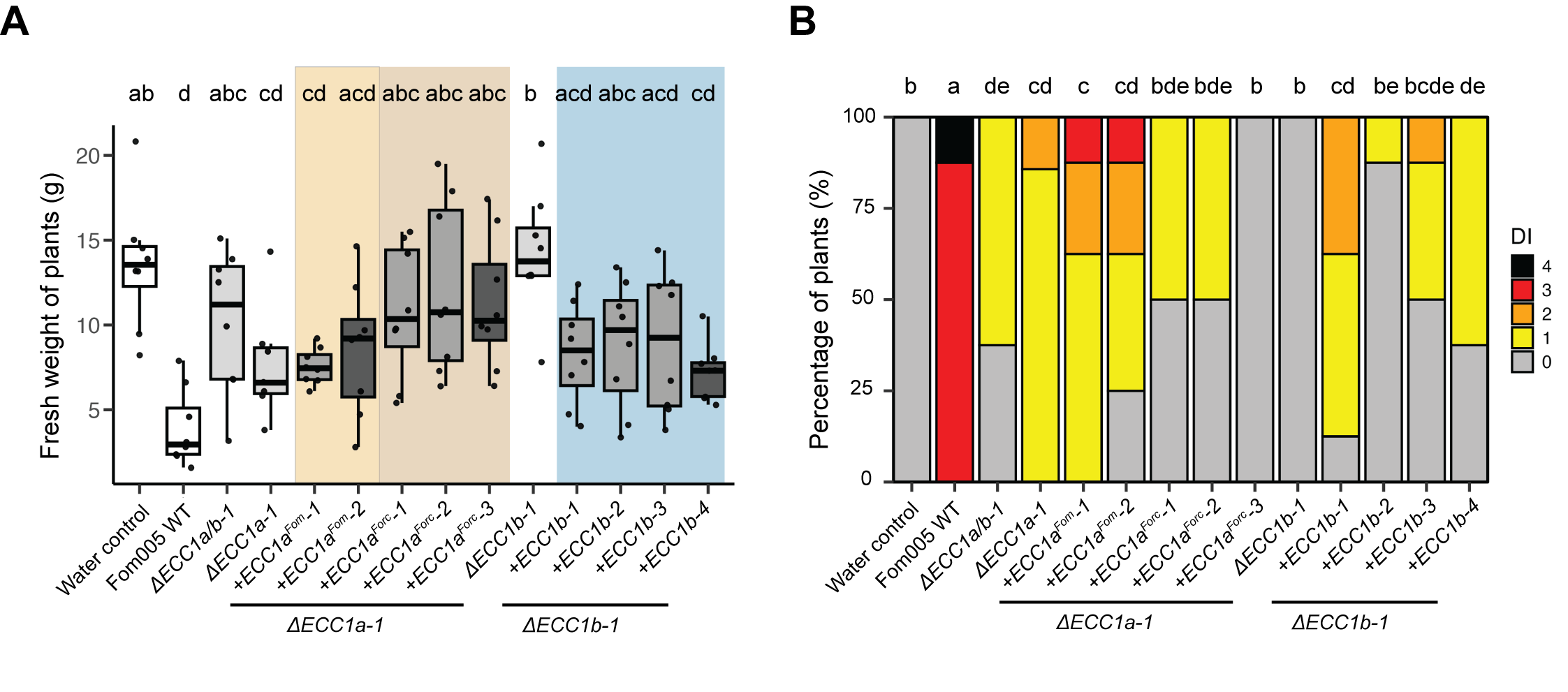

Supplement: Supplementary Figure 13 — ECC1 complementation partially restores virulence of Fom on melon and reveals contrasting roles in host specificity. Nine-day-old melon (Cucumis melo cv. Cha-T) seedlings were inoculated with water (mock), WT, ΔECC1a, ΔECC1b, ΔECC1a/b knockout mutants, complementation and gene replacement strains of Fom005 (n=8) at 25°C. (A) Plant fresh weight (FW) was measured (in grams) 14 days post inoculation. (B) Disease symptoms were scored 14 days post inoculation. Means followed by a common letter are not significantly different by Kruskal-Wallis with Dunn’s post hoc test and Benjamini-Hochberg correction for FW (A) or Mann-Whitney U test with Benjamini-Hochberg correction for disease symptoms (B) at the 5% level of significance. FW box plots are colored by strain type: mock/WT (white), knockouts (light grey), in locus complementation (medium grey), ectopic complementation (dark grey). [file Image13.tif]

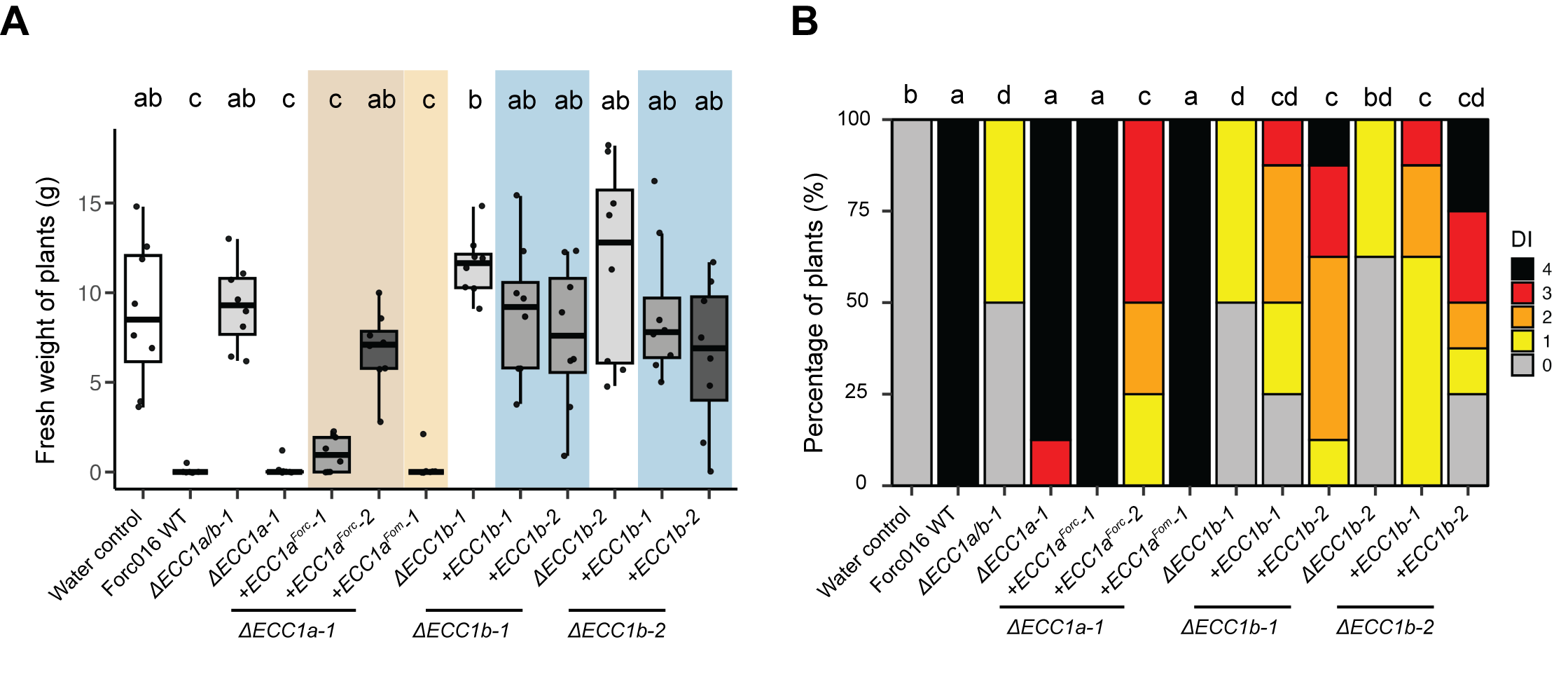

Supplement: Supplementary Figure 14 — ECC1aForc does not contribute to Forc virulence on melon. Nine-day-old melon (Cucumis melo cv. Cha-T) seedlings were inoculated with water (mock), WT, ΔECC1a, ΔECC1b, ΔECC1a/b knockout mutants, complementation and gene replacement strains of Forc016 (n=8) at 25°C. (A) Plant fresh weight (FW) was measured (in grams) 14 days post inoculation. (B) Disease symptoms were scored 14 days post inoculation. Means followed by a common letter are not significantly different by Kruskal-Wallis with Dunn’s post hoc test and Benjamini-Hochberg correction for FW (A) or Mann-Whitney U test with Benjamini-Hochberg correction for disease symptoms (B) at the 5% level of significance. FW box plots are colored by strain type: mock/WT (white), knockouts (light grey), in locus complementation (medium grey), ectopic complementation (dark grey). [file Image14.tif]
